# Unveiling reverse vaccinology and immunoinformatics toward Saint Louis encephalitis virus: a ray of hope for vaccine development

**DOI:** 10.3389/fimmu.2025.1576557

**Published:** 2025-05-19

**Authors:** Prasanna Srinivasan Ramalingam, Mahalakshmi Aranganathan, Md Sadique Hussain, Sujatha Elangovan, Gayathri Chellasamy, Purushothaman Balakrishnan, Janaki Ramaiah Mekala, Kyusik Yun, Sivakumar Arumugam

**Affiliations:** ^1^ Protein Engineering Lab, School of Biosciences and Technology, Vellore Institute of Technology, Vellore, TamilNadu, India; ^2^ School of Natural Sciences and Mathematics, The University of Texas at Dallas, Richardson, TX, United States; ^3^ Uttaranchal Institute of Pharmaceutical Sciences, Uttaranchal University, Dehradun, Uttarakhand, India; ^4^ Department of Bionanotechnology, Gachon University, Seongnam-si, Gyeonggi-do, Republic of Korea; ^5^ Department of Biomaterials, Saveetha Dental College and Hospitals, SIMATS, Saveetha University, Chennai, India; ^6^ Department of Biotechnology, Koneru Lakshmaiah Education Foundation, Green Fields, Guntur, Andhra Pradesh, India

**Keywords:** Saint Louis encephalitis virus, vaccine, epitope, antigen, immune response

## Abstract

**Introduction:**

Infectious diseases continue to challenge human health with high incidence and mortality rates worldwide. Notably, the adaptability of RNA viruses, highlighted by outbreaks of SARS, MERS, and COVID-19, emphasizes the timely need for effective therapeutics. Saint Louis encephalitis virus (SLEV) belonging to the Flaviviridae family is an RNA virus that mostly affects the central nervous system (CNS) of humans. Although supportive care treatments such as antiemetics and painkillers are being used against SLEV infection, it still lacks potential therapeutics for the effective treatment.

**Methods:**

Reverse vaccinology and immunoinformatics approaches help in the identification of suitable epitopes to design a vaccine construct that will activate both B- and T-cell-mediated responses. Previous studies used only the envelope protein E for the vaccine design, but we have used multiple protein targets to enhance the vaccine efficacy. Thus, in the present study, we have designed a multi-epitope subunit vaccine that specifically targets the membrane glycoprotein M, envelope protein E, and anchored capsid protein anchC of SLEV.

**Results:**

Our results indicated that the vaccine construct is structurally stable, antigenic, non−allergic, non−toxic, and soluble. Additionally, the vaccine construct was structurally refined and indicated significant binding affinity toward the Toll-like receptor 4 (TLR-4) supported by molecular docking and molecular dynamics simulations. Furthermore, it also indicated that it has the potential to induce an immune response.

**Conclusion:**

In addition, it has been cloned in the pET-28a (+) vector-6xHis-TEV-ORF9c expression vector for further experimental validation. We also recommend to evaluate the designed vaccine’s therapeutic efficacy through *in vitro* and *in vivo* studies in the near future.

## 1 Introduction

Infectious diseases caused by pathogenic microorganisms possess a significant challenge and health burden to humans with widespread morbidity and mortality worldwide (1–3). A recent report indicated that the frequency of SLEV varied from 80% of the cases in patients <20 years of age to 95% in those >60 years of age, and of the 47 confirmed human cases, 45 patients were hospitalized and among them 9 died at a younger age (4). Despite the advancements of several strategies to combat these pathogen-induced diseases, they adapt to extreme environments and even result in antimicrobial resistance (AMR) in the case of bacteria and antigenic shifts and drifts in the case of viruses (5–8). Additionally, urbanization (notably in low- to middle-income countries), globalization (rapid dissemination via travel), and sudden climate changes (high risk for outbreaks) also accelerated the wide spread of these infectious diseases causing localized outbreaks, widespread epidemics, and even global pandemics (9, 10). To note, the COVID-19 pandemic due to the SARS-CoV-2 outbreak resulted in high mortality and incidence rates and indicated the risk of these infectious diseases to human health (11–13). Furthermore, addressing these challenges requires multidisciplinary approaches such as a one-health approach, public health interventions, intensive medical research, systemic and bioinformatics approaches, and global collaborations to mitigate their impact on human health (14–16).

Saint Louis encephalitis virus (SLEV) belonging to the Flaviviridae family is a mosquito-borne flavivirus that has a single-stranded RNA in its genome (17, 18). SLEV is a zoonotic disease that is mainly transmitted from the bite of infected Culex mosquitoes, particularly *Culex pipiens*, *Culex quinquefasciatus*, and *Culex nigripalpus* and its first large endemic outbreak was observed in 1933 in the United States, and also observed in Central American and South American regions (18–21). However, Culex mosquitoes are not only restricted to the Americas; they have a global distribution and are commonly found in tropical and temperate regions worldwide, where they serve as vectors for multiple arboviruses including SLEV (20, 22). Primarily, birds are the reservoir hosts of SLEV and humans are the incidental and disease-obtaining hosts (17). SLEV is closely related to other flavivirus such as Japanese encephalitis (JEV) and West Nile virus (WNV), which often show asymptomatic conditions characterized by fatigue, headaches, nausea, vomiting, and body aches among the infected individuals (23–25). The cases of fatality rate for encephalitis caused by SLEV ranges from 5% to 15%, which mostly infects adults and could be diagnosed by neutralizing antibody testing and IgM ELISA kits (26–28). Unfortunately, there is no specific treatment available for SLEV-infected patients and potent prophylactic vaccines to combat SLEV infections; however, supportive care such as antiemetics and painkillers are being provided (26). Alongside, several therapeutic strategies are being developed and studied for the better treatment of SLEV in preclinical and clinical settings. Notably, two previous efforts were made to produce an SLEV vaccine. Hossain et al. have also employed the immunoinformatics approach to design a vaccine against SLEV and showed that it has potential against the envelope protein E SLEV, and Blaney Jr et al. have developed a live attenuated virus vaccine by employing SLE/DEN4-436,437 clone 41 and SLE/DEN4-654,655 clone 46 viruses (29, 30). To note, there is no vaccine currently available for the effective treatment of SLEV (28).

Reverse vaccinology and immunoinformatics approaches help in the identification of suitable epitopes to design a vaccine construct that will activate both B- and T-cell-mediated response using bioinformatics approaches (31). This approach has been extended toward the development of vaccines for various infectious diseases including SARS-CoV-2 and also extended to the development of cancer vaccines (32). In the present study, we employed reverse vaccinology and immunoinformatics approaches to design a multi-epitope subunit vaccine that specifically targets membrane glycoprotein M, envelope protein E, and anchored capsid protein anchC of SLEV.

## 2 Materials and methods

### 2.1 Data retrieval

Initially, Saint Louis encephalitis virus was provided as the query, and the FASTA sequences of the proteins, membrane glycoprotein M (NCBI Reference Sequence: YP_009329948.1), envelope protein E (NCBI Reference Sequence: YP_009329949.1), and the anchored capsid protein anchC (NCBI Reference Sequence: YP_009329944.1) of SLEV were retrieved from the NCBI-Protein database (https://www.ncbi.nlm.nih.gov/) (32, 33). The three-dimensional structures of the HLA-A*02:01 (PDB ID: 1DUZ), HLA-DRB1*01:01 (PDB ID: 1AQD), and Toll-like receptor 4 (TLR4) (PDB ID: 4G8A) were also retrieved from the Protein Data Bank (34, 35).

### 2.2 CTL and HTL epitope identification and selection

Since the cytotoxic T lymphocytes (CTL) (9-mer) and helper T lymphocytes (HTL) (15-mer) are involved in the induction of immune response in humans, the CTL and HTL epitopes were predicted using the NetCTL 1.2 web server (https://services.healthtech.dtu.dk/services/NetCTL-1.2/) and NetMHCII 2.3 web server (https://services.healthtech.dtu.dk/services/NetMHCII-2.3/), respectively (36, 37). For CTL epitopes, they were identified against the 12 types of MHC-I with 0.75 as the default threshold, and for the HTL epitopes, they were identified against all the alleles of HLA-DR, HLA-DQ, and HLA-DP, respectively. The robustness of the predictions was validated by ANN 4.0 and MHC Flurry 2.0 for MHC I epitopes and validated by Combinatorial library & Tepitope for MHC II epitopes in the IEBD tool, respectively (https://www.iedb.org/) (38). Following this, all the predicted epitopes were subjected to antigenicity (model set as tumor), allergenicity, and toxicity (SVM-based method) analysis using the VaxiJen v2.0 web server (https://www.ddg-pharmfac.net/vaxijen/VaxiJen/VaxiJen.html) (39), AllerTOP v.2 web server (https://www.ddg-pharmfac.net/allertop_test/) (40), and ToxinPred web server (https://webs.iiitd.edu.in/raghava/toxinpred/index.html) (41), respectively. Furthermore, the IFN-γ induction potential of HTL epitopes was also predicted with a hybrid approach (motif+SVM model) and the IFN-γ vs. non-IFN-γ model was used using the IFNepitope web server (https://webs.iiitd.edu.in/raghava/ifnepitope/application.php) (42). Alongside, the sequence conservation analysis of the predicted epitopes of SLEV was analyzed using protein-BLAST (https://blast.ncbi.nlm.nih.gov/Blast.cgi), toward Dengue virus 1 (taxid:11053), Zika virus (taxid:64320), Yellow fever virus (taxid:11089), West Nile virus (taxid:11082), and Japanese encephalitis virus (taxid:11072), which are closely related to the Flavivirus family.

### 2.3 Docking of T-cell epitopes with HLA alleles

The three-dimensional structures of the selected CTL and HTL epitopes were modeled using the PEP-FOLD 3.5 web server (https://bioserv.rpbs.univ-paris-diderot.fr/services/PEP-FOLD3/) (43). Furthermore, the CTL and HTL epitopes were docked against the HLA-A*02:01 and HLA-DRB1*01:01 alleles to evaluate their binding potential and molecular interactions against these more common alleles in the world population using the HPEPDOCK 2.0 web server (http://huanglab.phys.hust.edu.cn/hpepdock/) (44).

### 2.4 Vaccine construct design

The selected CTL epitopes, HTL epitopes, linkers, and adjuvant were used to design the multi-epitope vaccine construct. CTL epitopes were linked with the AAY linker and HTL epitopes with the CPGPG linker, whereas the adjuvant was connected with an EAAAK linker. The TLR4 agonist, 50s ribosomal L7/L12 protein of *Mycobacterium tuberculosis*, was used as an adjuvant in the vaccine construct to elucidate the strong immune response (45, 46).

### 2.5 Analysis of physicochemical characteristics, antigenicity, and allergenicity

The physicochemical properties such as the molecular weight, theoretical PI, amino acid composition and length, total number of negatively charged and positively charged residues, instability index, aliphatic index, and GRAVY of the designed multi-epitope vaccine were predicted using the Expasy ProtParam web server (https://web.expasy.org/protparam/) (47). In addition, the antigenicity, allergenicity, and solubility of the designed multi-epitope vaccine were predicted using the VaxiJen v2.0 web server (39), AllerTOP v.2 web server (40), and SOLpro web server (48), respectively. Furthermore, the antigenic nature of the adjuvant was predicted by evaluating the antigenicity of the designed multi-epitope vaccine with and without the presence of adjuvant using the ANTIGENpro web server (https://scratch.proteomics.ics.uci.edu/) (48).

### 2.6 Structural analysis and molecular docking of the designed vaccine construct

Initially, the 2D structure of the designed multi-epitope vaccine construct was predicted using the PDBsum database (http://www.ebi.ac.uk/thornton-srv/databases/pdbsum/Generate.html) (49). Then, the 3D structure of the designed multi-epitope vaccine construct was predicted using the I-TASSER web server (https://zhanggroup.org/I-TASSER/) (50) and further refined by the GalaxyRefine web server (https://galaxy.seoklab.org/cgi-bin/submit.cgi?type=REFINE) (51). In addition, the refined 3D model of the designed multi-epitope vaccine construct was validated by Ramachandran plot and Z-score plot by employing the PDBsum database and ProSA-web web server (https://prosa.services.came.sbg.ac.at/prosa.php), respectively (52). Then, the perfectly refined model was docked against the Toll-like receptor-4 (TLR4) protein using the ClusPro 2.0 web server (https://cluspro.bu.edu/login.php?redir=/home.php) (53).

### 2.7 Molecular dynamics simulations

The molecular dynamics simulations of the TLR4–vaccine complexes were performed using GROMACS 2020, and the protein topology files were generated using the GROMOS 42a1 force field (54, 55). The systems were solvated in an orthorhombic box using the simple point charge water model, and the neutralization was achieved by adding Na+ counter ions. Then, the energy minimization was carried out by the steepest descent algorithm with 50,000 steps, and the system was equilibrated under the NVT ensemble for 500 ps at 300 K, followed by NPT equilibration for 1,000 ps. Furthermore, the cutoff distance of 1.2 nm was applied for short-range non-bonded interactions, including Coulombic and van der Waals potentials, and the system was subjected to a 100-ns molecular dynamics simulation analysis. Finally, the resulting trajectories were analyzed to assess the root mean square deviation (RMSD), root mean square fluctuation (RMSF), radius of gyration (Rg), and solvent-accessible surface area (SASA) using standard GROMACS tools and visualized using the ggplot2 package (56, 57).

### 2.8 Normal mode analysis

The protein deformation analysis of the TLR4-vaccine docked complex was analyzed using the internal coordinates normal mode analysis (NMA) by employing the iMODS web server (https://imods.iqf.csic.es/) (58). The NMA analysis was conducted using the CA atomic model to evaluate their B-factor/mobility, eigenvalue, variance, covariance map, and elastic network for the TLR4-vaccine docked complex (59).

### 2.9 Immune response simulation

The immune response induction is a crucial factor in vaccination, and thus the immune response simulation of the designed multi-epitope vaccine construct was evaluated using the C-ImmSim web server (https://kraken.iac.rm.cnr.it/C-IMMSIM/index.php) that employs the position-specific score matrix (PSSM) and the Celada–Seiden model (60). The simulation parameters were configured with a random seed of 12,345, a simulation volume of 10 µL, and 1,095 simulation steps, representing a time span of 1 year (365 days). The vaccine was administered in three doses on days 0, 28, and 56, corresponding to time steps 1, 84, and 168, respectively. Injection modes were performed without LPS, and all other parameters were set to their default values.

### 2.10 Codon optimization and *in silico* cloning analysis

**Figure 1:**
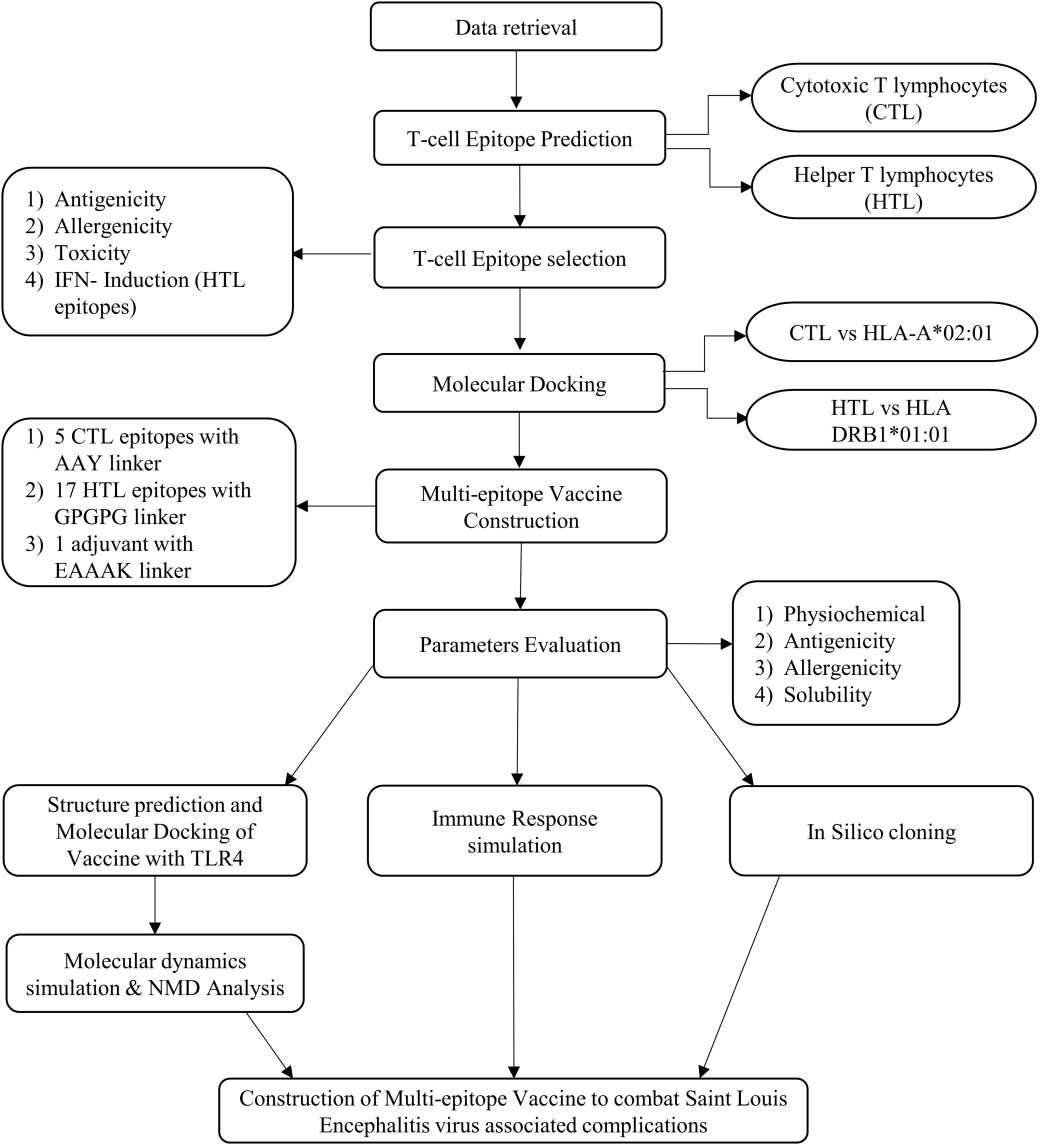
Schematic representation of the workflow of the study.

The vaccine construct’s protein sequence was reverse-translated, and its cDNA sequence was optimized for codon usage by employing the Java Codon Adaptation Tool (JCat) (https://www.jcat.de/), and the *E. coli* K12 was employed as the expression host (61). Then, the optimized sequence was inserted and cloned in the pET-28a (+) vector-6xHis-TEV-ORF9c (5,554 bp) using the SnapGene software (https://www.snapgene.com/). The complete schematic representation of the workflow of the study is shown in **Figure 1**.

## 3 Results

### 3.1 Selected T−cell epitopes showed potential interaction toward HLA alleles

The CTL epitopes (9-mer) and the HTL epitopes (15-mer) were predicted against the 12 types of MHC-I molecules, and all alleles of HLA-DR, HLA-DQ, and HLA-DP. The CTL epitopes predicted against the membrane glycoprotein M, envelope protein E, and anchored capsid protein anchC of SLEV along with their specific biding MHC-I allele are provided in **Supplementary Tables S1**, **S2**, and **S3**, respectively. Similarly, their predicted HTL epitopes of proteins of SLEV along with their specific biding MHC-II allele are provided in **Supplementary Tables S4**, **S5**, and **S6**, respectively. The antigenicity, allergenicity, and toxicity properties of the predicted CTL and HTL epitopes were evaluated, and IFN-γ induction potential was also predicted for the HTL epitopes (15mer). The epitopes were screened with these criteria such as antigenic, non-allergen, non-toxic, and IFN-γ induction (only for 15mer), and the shortlisted CTL and HTL epitopes are provided in **Supplementary Tables S7** and **S8**, respectively. The final CTL and HTL epitopes selected for the vaccine construct along with their epitope names are provided in **Supplementary Table S9**. Additionally, the sequence conservation analysis was performed toward Dengue virus 1 (taxid:11053), Zika virus (taxid:64320), Yellow fever virus (taxid:11089), West Nile virus (taxid:11082), and Japanese encephalitis virus (taxid:11072), which are closely related to the Flavivirus family, and the results are provided as similarity percentage in **Supplementary Table S9**. Notably, the West Nile virus and Japanese encephalitis virus shared a similarity percentage of most predicted epitopes, and Dengue virus 1 and Zika virus shared a similarity percentage with one CTL and one HTL epitope, respectively. This similarity-conserved epitopes have the potential to induce cross-reactive T-cell responses and broaden protection toward other species such as West Nile virus and Japanese encephalitis virus, indicating that the developed vaccine construct was broad-spectrum.

Furthermore, the selected CTL and HTL epitopes were docked against the HLA-A*02:01 and DRB1*01:01 alleles, which are the most frequent alleles among the world population, and their binding energies (kcal/mol) and docked pose are shown in **Table 1**, **Supplementary Figures S1** and **S2**. Totally, five CTL epitopes were docked against HLA-A*02:01 and 17 HTL epitopes were docked against HLA-DRB1*01:01 molecules. From the docking analysis, we observed that the CTL epitope (RVVFVIMLM) and the HTL epitope (TTQINYHWHKEGSSI) showcased high binding affinities toward their respective allele with binding energies of −240.708 and −234.422 kcal/mol, respectively, and the CTL epitope (TISPQAPSF) and HTL epitope (MKMEATELATVREYC) showcased comparatively less binding affinities toward their respective allele with binding energies of −210.309 and −182.066 kcal/mol, respectively. For CTL epitopes, the binding affinities range from −210.309 to −240.708 kcal/mol, and for HTL epitopes, they range from −182.066 to −234.422 kcal/mol. Moreover, all the CTL and HTL epitopes indicated their potential binding affinities and thus they were selected in the construction of a multi-epitope vaccine.

**Table 1:** Binding energies of selected CTL and HTL epitopes against HLA molecules.

Epitope type	Epitope	HLA molecule	Binding energy (kcal/mol)
CTL	NIKYEVAIF	HLA-A*02:01	−225.608
	TISPQAPSF	HLA-A*02:01	−210.309
	RDRSISLTL	HLA-A*02:01	−226.722
	QRVVFVIML	HLA-A*02:01	−213.502
	RVVFVIMLM	HLA-A*02:01	−240.708
HTL	ALAIGWMLGSNNTQR	HLA-DRB1*01:01	−216.101
	DFGSIGGVFNSIGKA	HLA-DRB1*01:01	−205.397
	GASGATWIDLVLEGG	HLA-DRB1*01:01	−194.994
	KMEATELATVREYCY	HLA-DRB1*01:01	−215.410
	LFGGMSWITQGLLGA	HLA-DRB1*01:01	−233.383
	LGALLLWMGLQARDR	HLA-DRB1*01:01	−227.230
	LVTVNPFISTGGANN	HLA-DRB1*01:01	−223.634
	MKMEATELATVREYC	HLA-DRB1*01:01	−182.066
	MSWITQGLLGALLLW	HLA-DRB1*01:01	−220.584
	NLPWTSPATTDWRNR	HLA-DRB1*01:01	−232.190
	PQAPSFTANMGEYGT	HLA-DRB1*01:01	−207.506
	PTLDFKVMKMEATEL	HLA-DRB1*01:01	−227.681
	REYCYEATLDTLSTV	HLA-DRB1*01:01	−206.449
	SGINTEDYYVFTVKE	HLA-DRB1*01:01	−240.228
	TKQTVVALGSQEGAL	HLA-DRB1*01:01	−201.193
	TTQINYHWHKEGSSI	HLA-DRB1*01:01	−234.422
	TIDCEARSGINTEDY	HLA-DRB1*01:01	−188.060

### 3.2 Designed multi-epitope vaccine showed desired physiochemical properties

**Figure 2:**
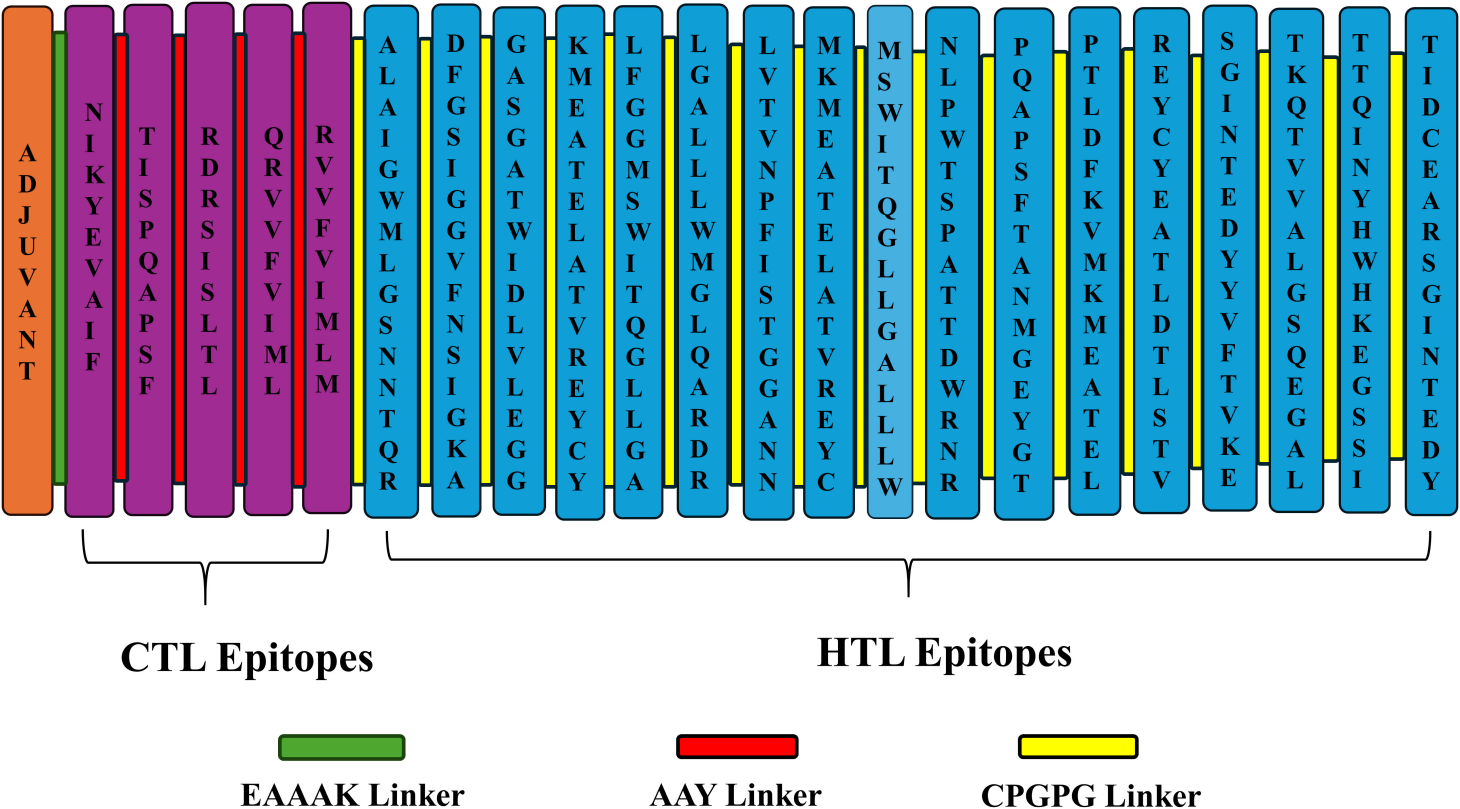
Pictorial representation of the designed multi-epitope vaccine construct.

**Figure 3:**
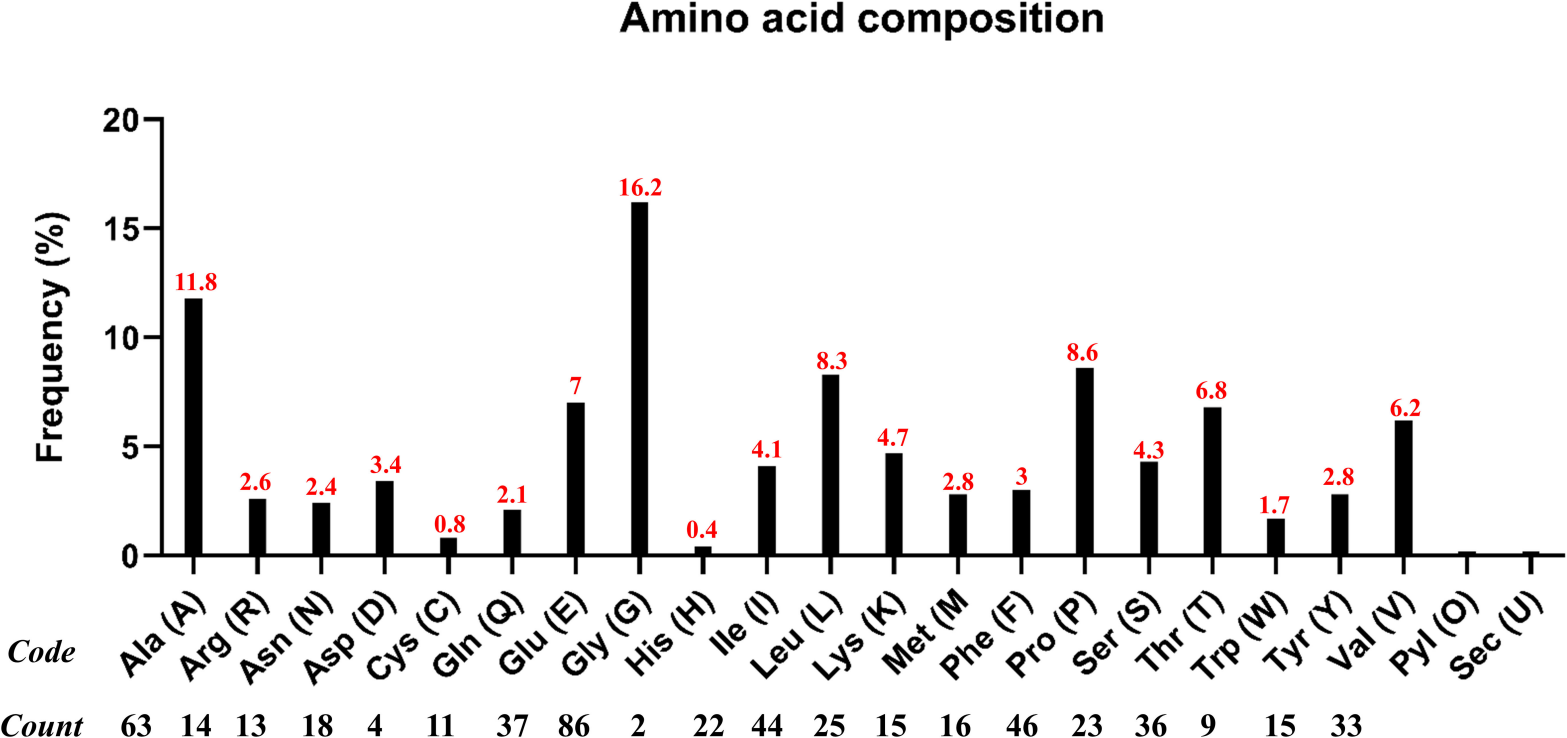
Amino acid composition and frequency of the designed multi-epitope vaccine construct.

Generally, adjuvants are used in multi-epitope peptide vaccines to induce strong immune responses when injected into humans. In our study, we have used the C-terminal region of the large ribosomal subunit protein bL12 of *Mycobacterium tuberculosis* as the adjuvant (MAKLSTDELLDAFKEMTLLELSDFVKKFEETFEVTAAAPVAVAAAGAAPAGAAVEAAEEQSEFDVILEAAGDKKIGVIKVVREIVSGLGLKEAKDLVDGAPKPLLEKVAKEAADEAKAKLEAAGATVTVK), which highly prevents the autoimmune reactions. AAY linkers were used to link the CTL epitopes, GPGPG linkers were used to link the HTL epitopes, and the EAAAK linker was used to link the adjuvant in the vaccine construct. The total vaccine construct contains 532 amino acids, comprising 5 CTL epitopes, 17 HTL epitopes, 1 adjuvant, 4 CTL linkers, 16 HTL linkers, and 1 adjuvant linker, as shown in **Figure 2**. Following this, the physiochemical properties of the designed multi-epitope vaccine construct were evaluated and are tabulated in **Table 2**. We observed that alanine (A) is more frequent with 11.8% followed by Arg (R) with 2.6%, as shown in **Figure 3**. The SOL-pro web server indicated the soluble nature of the designed multi-epitope vaccine construct with a probability of 0.902, and the instability index of 23.84 (less than 40) indicates the stability of the vaccine. The antigenic score of the designed multi-epitope vaccine construct was observed to be 0.787234 (without adjuvant) and 0.898972 (with adjuvant), indicating the increase in antigenic response when adjuvant is added to the vaccine construct. The Grand Average of Hydropathy (GRAVY) is used to determine the hydrophobic nature of the protein and is generally calculated by summing up the hydropathy values of all the amino acids and dividing it by the total number of amino acids of the protein. The positive value indicates the hydrophobic nature and the negative value indicates the hydrophilic nature of the given protein. In our study, the vaccine construct showed a GRAVY score of −0.040 that indicates its hydrophilic nature, as shown in in **Table 2**.

**Table 2:** Physiochemical properties of the designed vaccine construct.

Parameter	Value/range
Number of amino acids	532
Molecular formula	C_2440_H_3808_N_636_O_741_S_19_
Molecular weight	54518.03 Da
Theoretical pI	4.80
Total number of positive charge residues (Arg + Lys)	55
Total number of negative charge residues (Asp + Glu)	39
Instability index	23.84
Aliphatic index	78.21
GRAVY	−0.040
Estimated half life	30 h (mammalian reticulocytes, *in vitro*). >20 h (yeast, *in vivo*). >10 h (Escherichia coli, *in vivo*).

### 3.3 Structural modeling and refinement of the multi-epitope vaccine

**Figure 4:**
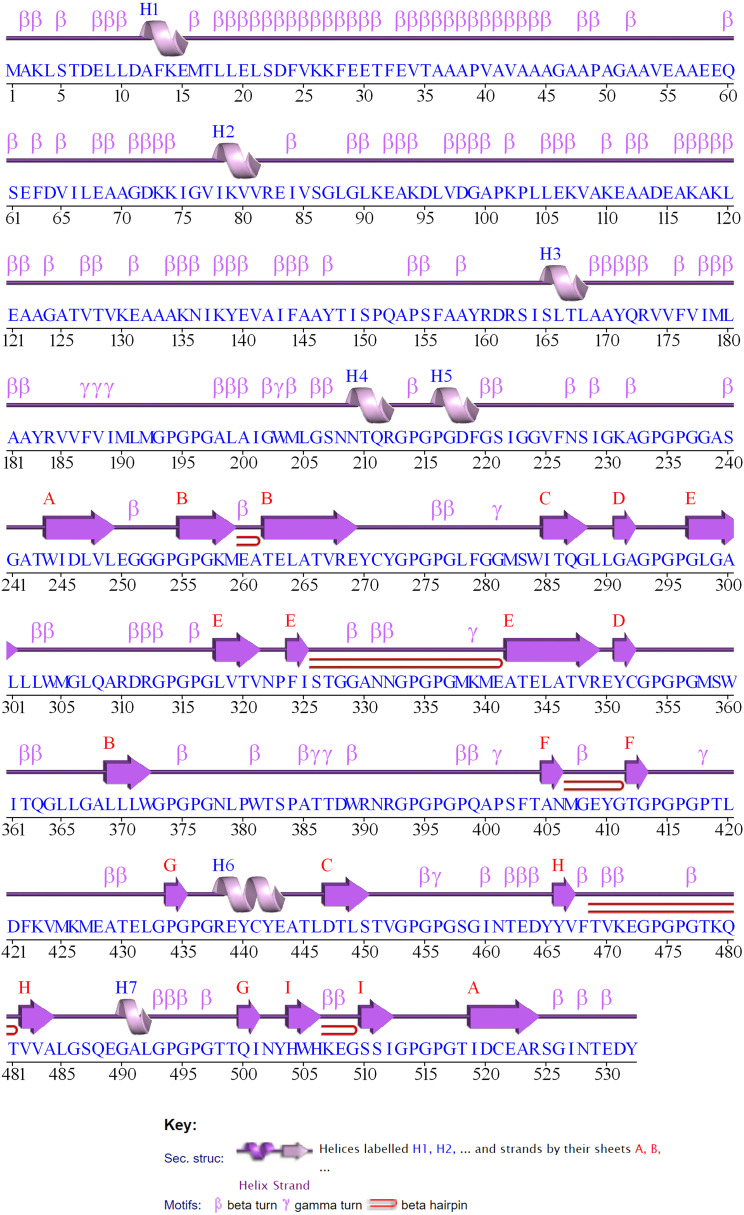
2D structure of the designed multi-epitope vaccine construct.

**Figure 5:**
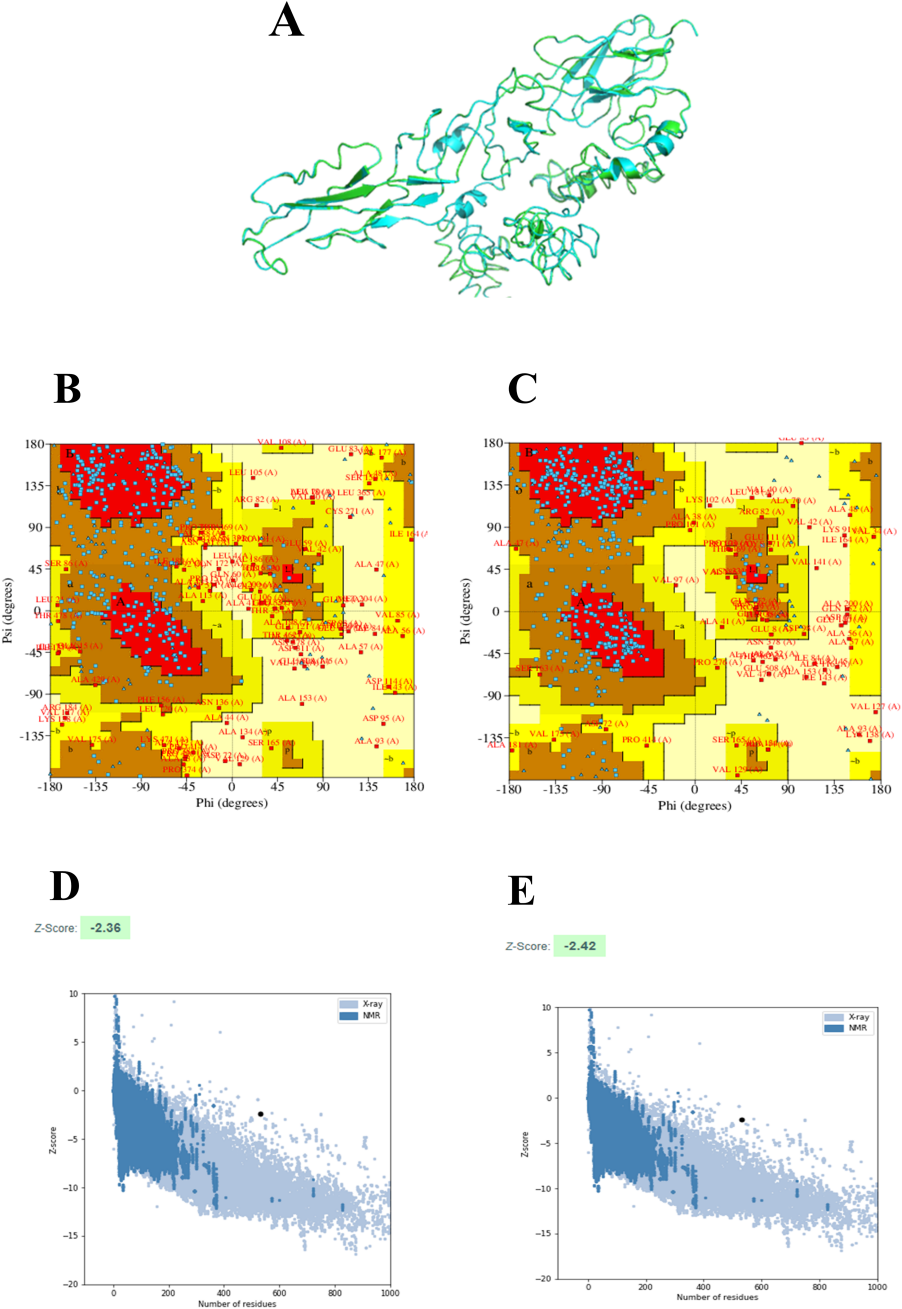
3D structure-refinement and validation: 3D structure of the designed multi-epitope vaccine construct in which the refined and unrefined models are shown in green color and cyan color, respectively **(A)**. Ramachandran plots of the unrefined **(B)** and refined models **(C)**. Z-score of the unrefined **(D)** and refined models **(E)**.

The 2D structure of the designed multi-epitope vaccine construct consisting of 532 amino acids was predicted and observed to have 8 sheets, 5 beta hairpins, 2 beta bulges, 19 strands, 6 helices, 181 beta turns, and 36 gamma turns, as shown in **Figure 4**. Then, the 3D structure was modeled by the I-TASSER web server, which resulted in five best models with C-scores of −3.15, −3.58, −3.63, −3.79, and −3.68, respectively. Generally, the high C-score represents the high confidence of the predicted model, and thus model 1 with a c-score of −3.15 was selected for further refinement acknowledging the crucial role of accurate 3D structural prediction in understanding the vaccine’s potential efficacy and stability. Likewise, the GalaxyRefine web server resulted in the best five refined models, in which model 2 was chosen based on a comprehensive evaluation of several structural parameters: a high GDT-HA score of 0.8459, a low RMSD of 0.707, a favorable MolProbity score of 3.467, a clash score of 78.9, a low percentage of poor rotamers at 2.1%, and a significant proportion of Ramachandran favored regions at 67.9% as shown in **Figure 5A**. These metrics collectively suggest a highly refined and accurate model, crucial for ensuring the vaccine’s effectiveness and structural refinement. Furthermore, the refined model models were validated by Ramachandran plot and Z-score analysis. The most favored regions on a Ramachandran plot are important because they help to identify the validity of a vaccine construct’s 3D structure and indicate which Phi/Psi angles are possible for an amino acid; thus, high % of most favored regions indicates better structural enhancement whereas the less % shows poor enhancement. Notably, in our findings, the Ramachandran plot analysis demonstrated an increase in the most favored regions from 43.2% in the unrefined model to 56.8% in the refined model, indicating improved structural quality and reduced steric clashes. Also, the G-factor, measuring the overall structural unusualness, improved significantly from −2.02 (unrefined) to −1.24 (refined), highlighting the enhanced accuracy and reliability of the refined model, as depicted in **Figures 5B** and **C**, respectively. In the Z-score plot, the higher negative value indicates the high confidence of the modeled structure of the vaccine construct whereas the lesser negative value indicates less confidence and the positive value indicates very poor confidence of the vaccine structure. In our study, we have observed that the Z-score was improved from −2.36 (unrefined) to −2.42 (refined), further confirming the high structural refinement and enhanced stability of the vaccine construct, as shown in **Figures 5D** and **E**, respectively.

### 3.4 Designed multi-epitope vaccine showed significant binding affinities toward TLR4

**Figure 6:**
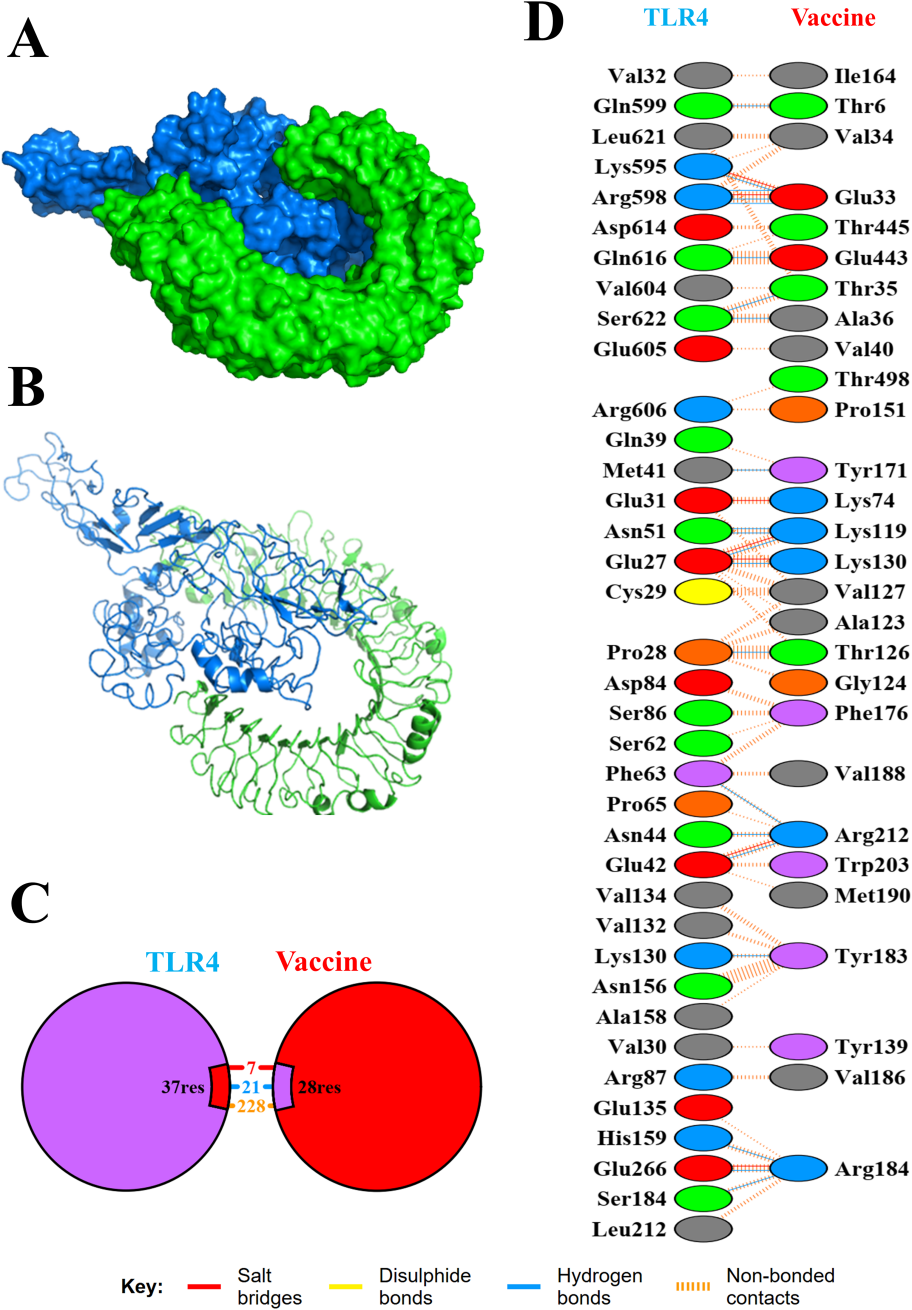
Molecular interaction of TLR4 with the designed multi-epitope vaccine construct. The docked complexes are shown in cartoon model **(A)** and surface model **(B)**. Also, total numbers of interactions **(C)** and interacting residues **(D)** of the TLR-4 vaccine complex are shown.

**Figure 7:**
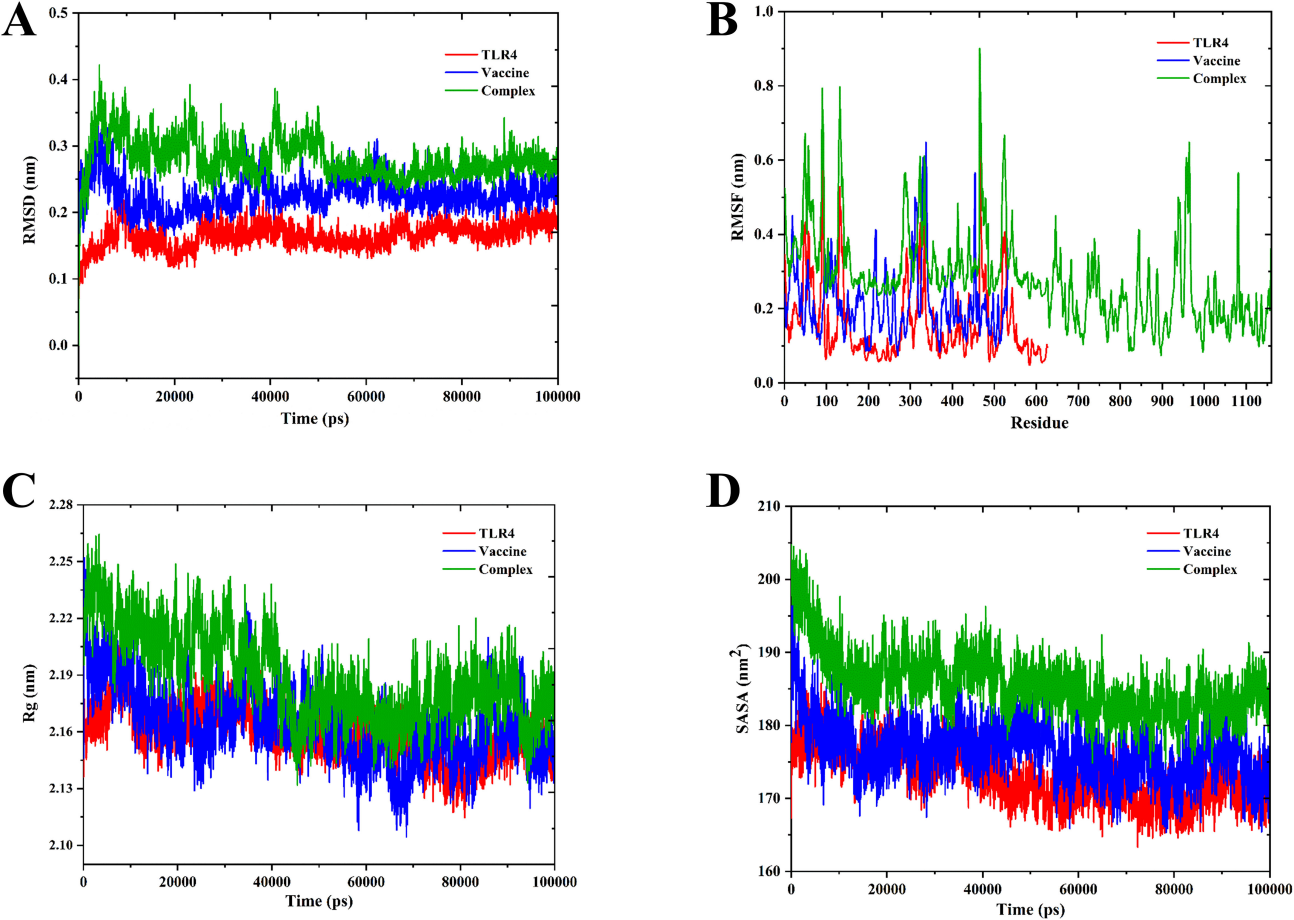
Molecular dynamics simulation of TLR4, vaccine, and docked complexes. The RMSD **(A)**, RMSF **(B)**, Rg **(C)**, and SASA **(D)** plots of TLR4, vaccine, and docked complexes are shown in red, blue, and green colors, respectively.

The binding affinity of the multi-epitope vaccine construct toward the Toll-like receptor-4 (TLR4) was evaluated using the ClusPro 2.0 web server, which generated nearly 29 clusters of potential docked conformations, and the cluster 17 for its best conformations of the docked complex. The binding energy of the multi-epitope vaccine construct and TLR4 docked complex was observed to be −1,117.5 kcal/mol, indicating the high binding affinity and favorable interaction between TLR4 and the multi-epitope vaccine construct. Upon binding, the vaccine showed a 1,762-Å^2^ interface area with 28 interacting residues and the TLR4 showed a 1,671-Å^2^ interface area with 37 interacting residues. Also, it revealed that it formed 7 salt bridges, 21 H-bonds, and 228 non-bonded contacts, as shown in **Figure 6**. Then, the molecular dynamics simulation trajectories were analyzed to study the conformational behavior of the TLR, vaccine, and TLR–vaccine complex over 100 ns. RMSD values were used to assess the local flexibility of the proteins, reflecting their atomic mobility. Higher RMSD values indicate increased mobility, whereas lower values suggest greater structural stability. During the simulation, the average RMSD values for the TLR, vaccine, and TLR–vaccine complex were 0.16, 0.22, and 0.28 nm, respectively. Then, RMSF plots revealed that TLR4 had fluctuations at the 120–170 AA and 310–320 AA regions, vaccine had fluctuations at the 320–325 AA region, and the TLR4–vaccine docked complexes exhibited the same fluctuations; however, these regions are denoted as loop regions. The average Rg values for the TLR, vaccine, and TLR–vaccine complex were 2.15, 2.16, and 2.18 nm, respectively, exhibiting the compactness of the structures. The average SASA values for the TLR, vaccine, and TLR–vaccine complex were 173, 176, and 186 nm², respectively, as shown in **Figure 7**.

### 3.5 NMA of the multi-epitope vaccine construct

**Figure 8:**
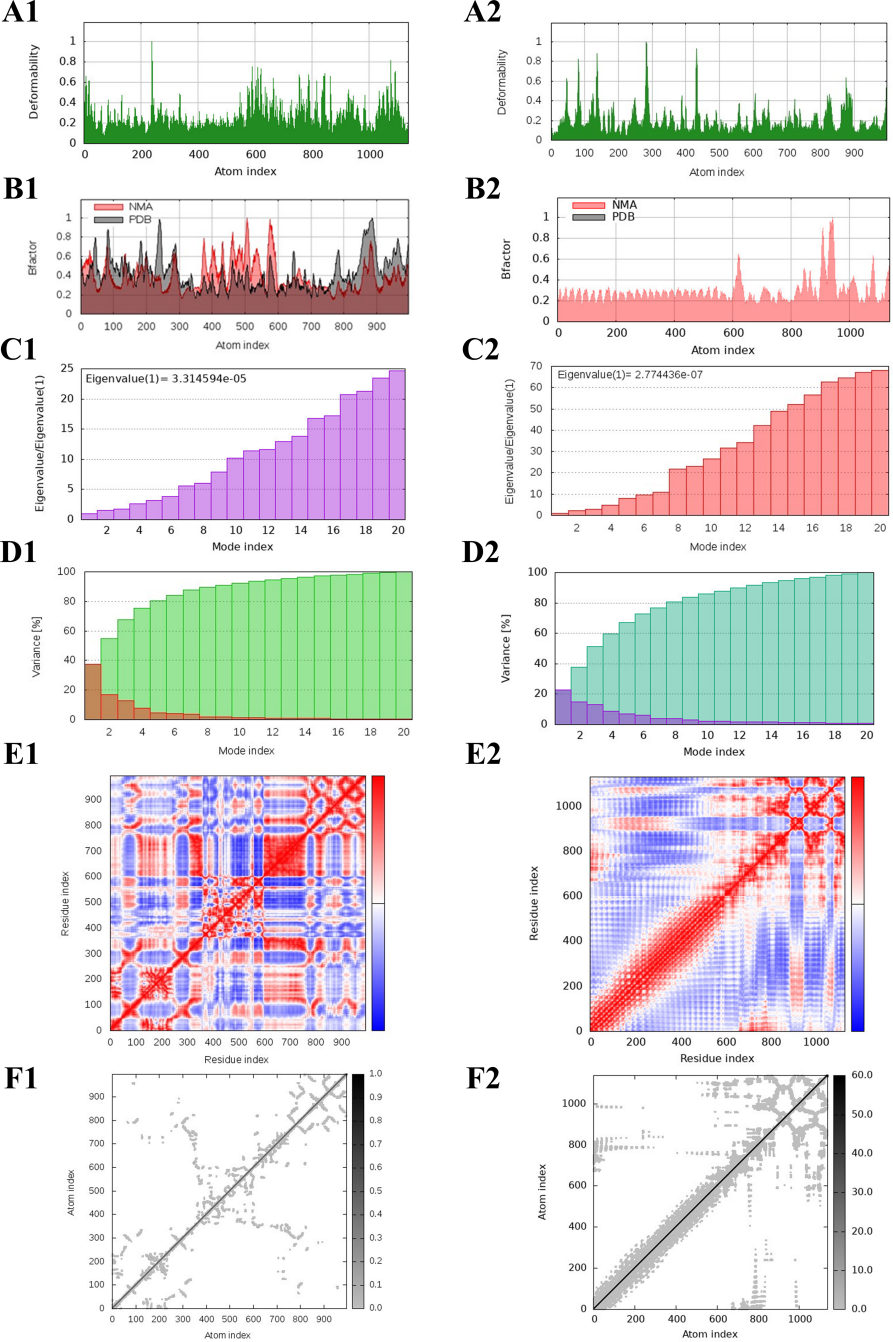
NMA of TLR4 and the designed multi-epitope vaccine construct–TLR4 docked complex. The deformability, B-factor, eigenvalues, variance, co-variance, and elastic network of the TLR4 **(A1-F1)** and TLR4–vaccine docked complex **(A2-F2)** are shown.

The protein deformation analysis of the multi-epitope vaccine construct and TLR4 docked complex was predicted as normal mode analysis (NMA). The flexibility and stability of the docked complexes were evaluated from various plots such as B-factor/mobility, eigenvalue, variance, and co-variance map of the elastic network of the TLR4–vaccine complex, as illustrated in **Figure 8**. The B-factor/mobility indicates less deformation of the TLR4–vaccine complex at all amino acid residues and hinges, indicating that it maintains structural integrity. Notably, a lower eigenvalue of 2.77e−07 indicates less deformability of the docked complex, than the TLR4 alone, which showed an eigenvalue of 3.31e−05. In addition, the individual and cumulative variances indicate the contribution of each normal mode to the overall motion. The co-variance map revealed the presence of correlated, uncorrelated, and anti-correlated residue pairs, providing insights into the cooperative movements within the complex. Furthermore, flexibility was also observed from the elastic network.

### 3.6 Designed multi-epitope vaccine has the potential to induce immune response

**Figure 9:**
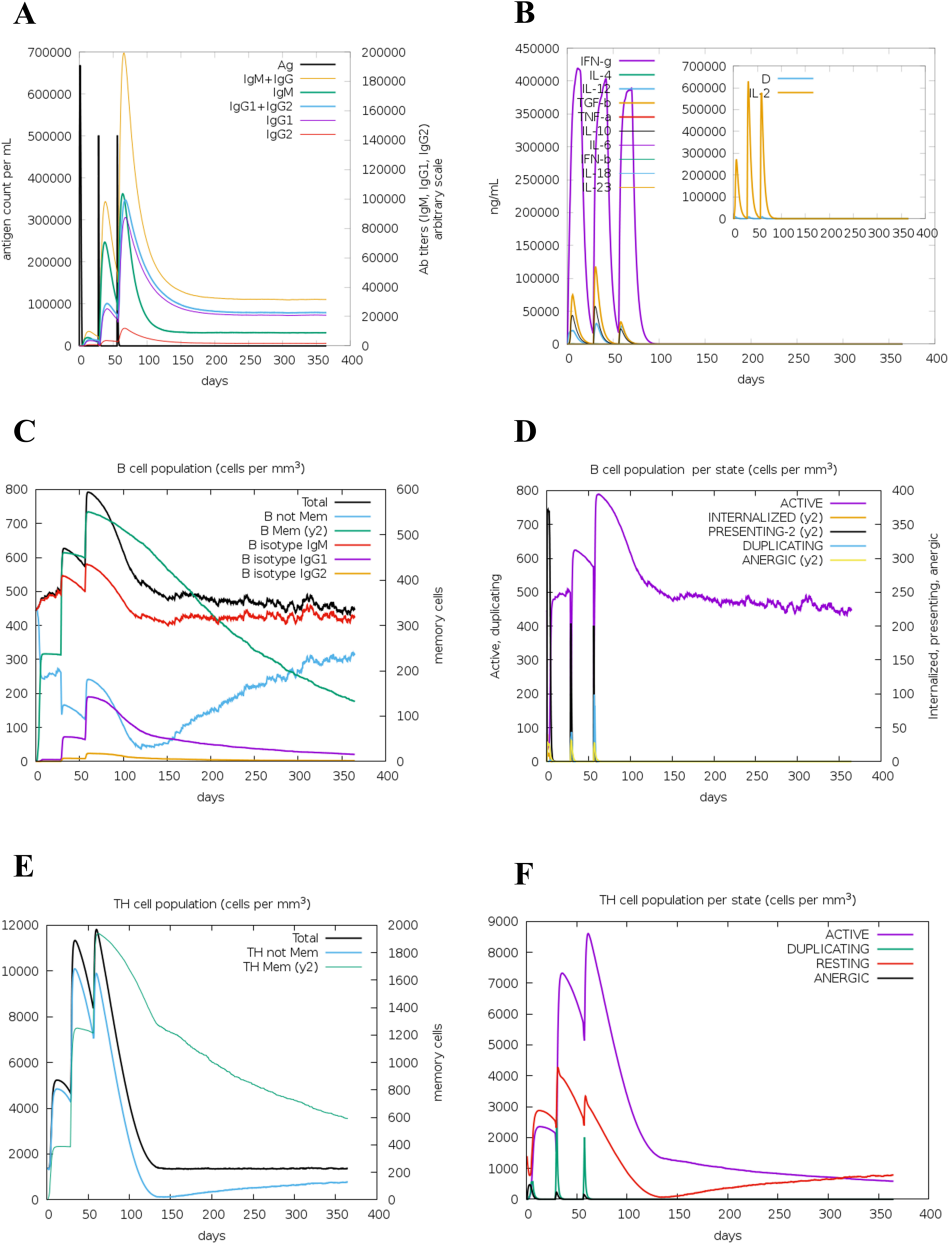
Immune simulation of the multi-epitope vaccine construct. Antibody titer **(A)**, cytokine production **(B)**, B-cell population **(C)**, B-cell population per state **(D)**, TH-cell population **(E)**, and TH-cell population per state **(F)** are shown.

The immune response simulation of the designed multi-epitope vaccine construct was predicted using the C-ImmSim web server at three dosage days. The immunological parameters such as the antibody titers, cytokine production, B-cell populations, B-cell populations per state, TH-cell populations, and TH-cell populations per state were predicted as shown in **Figure 9**. In the antibody titers plot, we have observed that IgG and IgM are significantly increased post-vaccine injection, indicating a robust humoral immune response. Also, the cytokine levels of IFN-γ were elevated notably, which suggested a strong activation of cellular immunity. Furthermore, the B-cell population (cells/m³) was elevated, reflecting the activation and proliferation of B cells in response to the vaccine. The total TH-cell population (cells/m³) also showed an increase, indicating enhanced helper T-cell responses, with a significant proportion of TH cells in active states, further corroborating the vaccine’s efficacy. Notably, all the predicted parameters such as the antibody titers (IgG and IgM), cytokine production (IFN-γ, ILs), B-cell populations (Total), B-cell populations per state (active state), TH-cell populations (Total), and TH-cell populations per state (active state) showed elevated peaks at the vaccine dosage days, indicating that the designed vaccine construct is highly efficient in inducing the immune responses in a time-dependent manner. These findings highlight the potential effectiveness of the multi-epitope vaccine construct in eliciting a comprehensive immune response, demonstrating its ability to induce both humoral and cellular immunity, which is crucial for long-term protection and memory formation.

### 3.7 Codon optimization and *insilico* cloning of the multi-epitope vaccine construct

**Figure 10:**
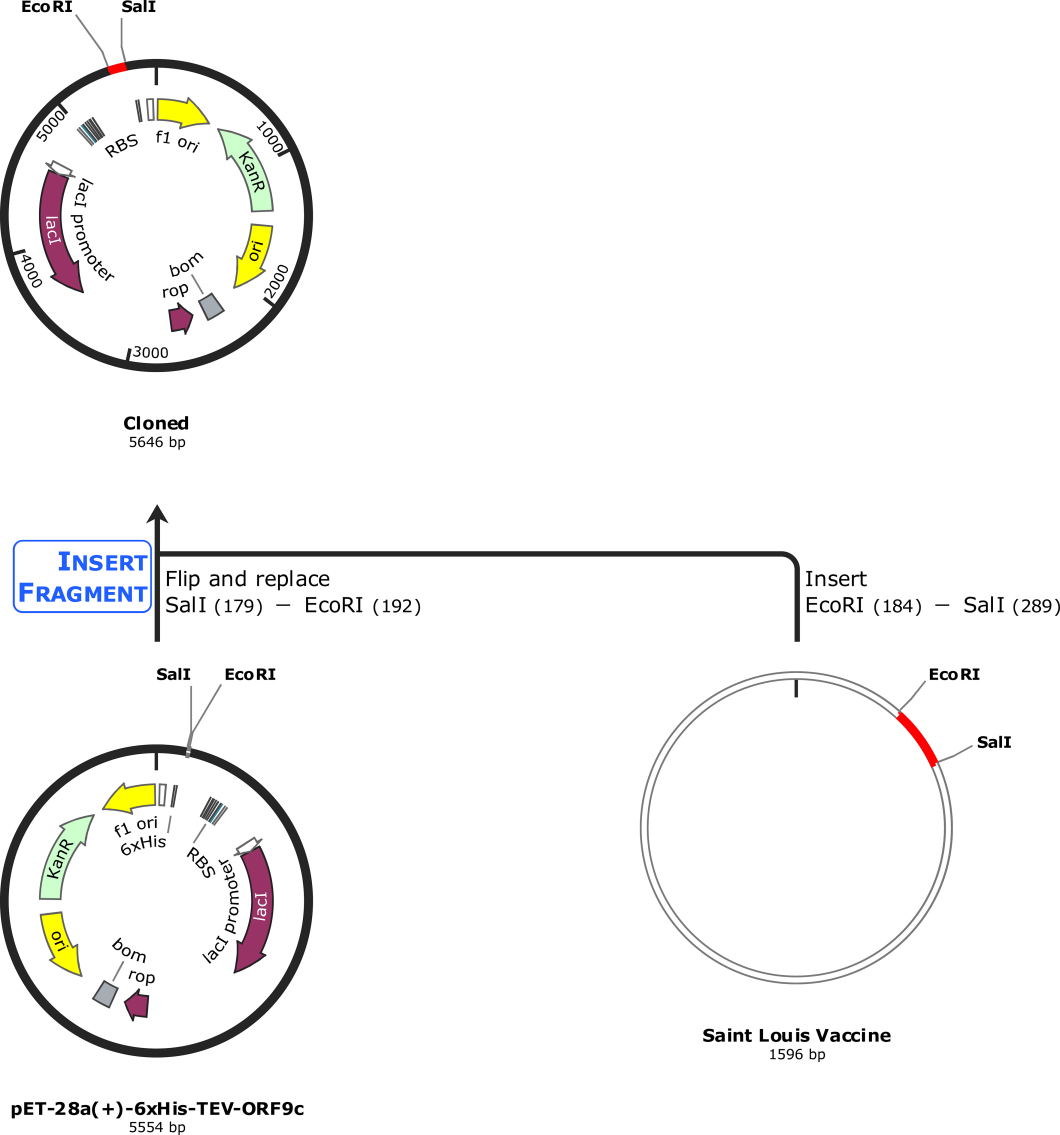
*In silico* cloning of the optimized vaccine construct. The codon-optimized multi-epitope vaccine sequence shown in red was cloned in the pET-28a (+) expression vector (5,554 bp) shown in black between restriction sites SalI and EcoRI, and the final cloned vaccine construct is shown (5,646 bp).

The designed multi-epitope vaccine construct was further reverse-translated and optimized to be cloned by employing *Escherichia coli* K12 as an expression system. Then, the optimized sequence containing 552 nucleotides was obtained with the CAI-value as 1, and GC% as 53.8%. Also, the GC% of *E. coli* strain K12 was observed as 50.73%. Then, the restriction sites of SalI (GAGCTC) and EcoRI (GAATTC) were added at the N-terminal and C-terminal of the optimized DNA sequence. Following this, the optimized vaccine construct sequence (564 nucleotides) was cloned into the pET-28a (+) vector-6xHis-TEV-ORF9c (5554 bp) at restriction sites of SacI (GAGCTC) and EcoRI (GAATTC) using the SnapGene tool, and the final cloned product (5646 bp) is shown in **Figure 10**.

## 4 Discussion

Infectious diseases have posed significant challenges to human health throughout history, manifesting in acute, chronic, and often lethal forms caused by various pathogenic microorganisms with widespread morbidity and mortality worldwide (1, 3). The emergence of antimicrobial resistance (AMR) and antigenic shifts and drifts challenge our advances in the medical field (62, 63). The recurring outbreaks of SARS, MERS, and COVID-19 underscore the adaptive potential of RNA viruses, which can mutate to exploit new niches (11, 64). SLEV infection is strongly associated with potential central nervous system impairment that highly targets adults and still lacks potential treatment strategies (17). Alongside, the peptide vaccines constructed with multiple epitopes have recently gained attention due to their ability to amplify immune responses against pathogens (65). Both B cells and T cells can be used for vaccine development, but mostly T cell-based vaccines are preferred to some reasons such as high specificity adaptive immunity. CD8+ T cells uniquely recognize and eliminate infected cells via MHC, long-term immune memory, and broader immunological coverage. Notably, for viral infections that have antigenic variation, the B cell-mediated antibody responds less effectively (66). Although the traditional vaccines have the potency to induce strong humoral, cellular responses, and need fewer boosters than peptide vaccines, they are limited by their stability, risk of reversion to virulence, allergic reactions, and live-attenuated rapid mutation rates that lead to low efficacy in immune-compromised patients (67). On the other hand, the peptide vaccines are made of epitopes that specifically induce the stimulation of CTLs, HTLs, or B cells and have minimum off-target effects indicating low adverse reactions. Also, peptide vaccines are easy to design, produce, and store; cost-effective; and, mostly importantly, safer for immunocompromised individuals (68, 69). Hossain et al. have also employed the immunoinformatics approach to design a vaccine against SLEV and showed that it has potential against SLEV. However, they have predicted the multi-epitopes only for the envelope protein E (outer membrane protein) (29). In addition, to the best of our knowledge, this is the only report we found for employing reverse vaccinology and immunoinformatics to design multi-epitope vaccine construct against SLEV. Thus, we have designed the multi-epitope vaccine construct toward various key proteins of SLEV such as the membrane glycoprotein M, envelope protein E, and the anchored capsid protein anchC to increase its therapeutic potential in SLEV treatment. So, in the present study, we have designed and developed a multi-epitope vaccine construct against the SLEV by employing reverse vaccinology and immunoinformatics approaches.

The cytotoxic T lymphocytes (CTLs) (CD8+ T cell epitopes) are involved in the recognition, direct killing, and clearance of the virally infected cells, whereas the helper T lymphocytes (HTLs) (CD4+ T cell epitopes) are involved in the immune activation, antibody production, and cytokine secretion respectively, and thus they play a vital role in the vaccine design (70, 71). Also, to elucidate a proper immune response, the epitopes should be antigenic, non-allergenic, and non-toxic and have the potential to induce IFN-γ (HTL epitope) production (72, 73). In our study, we have predicted the possible CTL and HTL epitopes against the various key proteins of SLEV such as the membrane glycoprotein M, envelope protein E, and the anchored capsid protein anchC, and we have selected 5 CTL epitopes and 17 HTL epitopes based on the abovementioned criteria, as shown in **Supplementary Table S9**. Additionally, the sequence conservation analysis was performed toward Dengue virus 1 (taxid:11053), Zika virus (taxid:64320), Yellow fever virus (taxid:11089), West Nile virus (taxid:11082), and Japanese encephalitis virus (taxid:11072), which are closely related to Flavivirus family. Notably, the West Nile virus and Japanese encephalitis virus shared similarity percentages of most predicted epitopes, and Dengue virus 1 and Zika virus shared similarity percentages with 1 CTL epitope and 1 HTL epitope. This similarity-conserved epitopes have the potential to induce cross-reactive T-cell responses and broaden protection toward other species such as West Nile virus and Japanese encephalitis virus, indicating that the developed vaccine construct was broad-spectrum (74). HLA-A*02:01 (MHC-I) and DRB1*01:01 (MHC-II) are the most frequently expressed alleles that could bind with CTL and HTL epitopes, respectively (75, 76). For instance, HLA-A*02:01 belongs to the A2 supertype possesses supertypic representation, which covers multiple related alleles, expanding their population coverage, whereas HLA-DRB1*01:01 is immunodominant, binds a broad spectrum of peptides, and significantly elicits CD4+ T-cell responses (76, 77). We observed that the selected CTL and HTL epitopes exhibited significant binding affinities toward their respective allele and their binding energy was predicted as shown in **Table 1**. Unlike the mRNA vaccines, the peptide vaccines have the advantage of adding adjuvants along with the peptides, which could induce more antigenic-mediated immune responses (78, 79). We have utilized the C-terminal region of the large ribosomal subunit protein bL12 of *Mycobacterium tuberculosis* as the adjuvant, which highly prevents the autoimmune reactions.

The linkers play a vital role in the designing of the vaccine construct to elucidate proper structural and functional properties. The EAAAK linker elevates the antigenic nature of the vaccine, the AAY linker promotes the presentation of antigens, and the GPGPG linker promotes solubility and movement (78, 80, 81). Likewise, the adjuvant was connected with EAAAK linkers, CTL epitopes were connected with AAY linkers, and the HTL epitopes were connected with GPGPG linkers, and the designed multi-epitope vaccine construct comprising 532 amino acids has 5 CTL epitopes, 17 HTL epitopes, 1 adjuvant, 4 CTL linkers, 16 HTL linkers, and 1 adjuvant linker, as shown in **Figure 2**. The designed vaccine should be stable to have a longer half-life period and immunogenicity retention and avoid degradation, and should be soluble to have an enhanced bioavailability nature, to prevent aggregation and for efficient delivery (82, 83). Based on these criteria, several vaccines have been designed and developed against various diseases and infections (31, 84–86). Similarly, we have observed that our designed multi-epitope vaccine construct was soluble with a probability of 0.902 and stable with an instability index of 23.84 (less than 40) indicating the stability of the vaccine. Also, we observed that the addition of adjuvant increased the vaccine’s antigenic nature from 0.787234 to 0.898972, as shown in in **Table 2**.

Structural properties of the vaccine alter its functional properties such as the antigen presentation and stimulation of T lymphocytes and B lymphocytes (73). We have predicted the 3D structure of the designed vaccine construct and further refined it. Furthermore, we validated by the Ramachandran plot that showed highly favored regions in the refined model, and by the Z-score that showed high confidence in the refined 3D model. These analyses underscore the critical improvements in structural prediction and refinement processes, ensuring the vaccine construct’s robustness and potential efficacy. The refined model’s superior quality and stability are indicative of its potential to elicit a strong and effective immune response, thereby validating its design and functional applicability (87). Unlike the other Toll-like receptors (TLRs), Toll-like receptor-4 is observed to be overexpressed and also involved in various functions such as promoting the production of pro-inflammatory cytokine and chemokine and regulation of homeostasis, and thus plays a vital role in various diseases including SLEV infection (87, 88). Thus, we have docked our vaccine construct with TLR4, which showed significant binding affinities with a binding energy of −1,117.5 kcal/mol. The low binding energy profile suggests that the multi-epitope vaccine construct is likely to form a stable and effective complex with TLR4, potentially enhancing its immunogenic efficacy and contributing to a robust immune response. The molecular dynamics simulation (MDS) revealed that the TLR4-vaccine docked complex was stable throughout the simulation period compared with TLR4 and vaccine alone, indicating the structural compatibility of the docked complex as shown in **Figure 7**. Also, it indicated that there is no flip on the residues of the TLR4–vaccine complex confirmed through MDS. Additionally, the TLR4–vaccine docked complex was also observed to be stable through the protein deformation analysis evaluated from various plots such as B-factor/mobility, eigenvalue, variance, and co-variance map of the elastic network of the TLR4–vaccine complex, as illustrated in **Figure 8**.

Generally, the vaccine-induced immune response is crucial, and multifaceted, encompassing both innate and adaptive immunity (89, 90). From our study, we observed that the designed multi-epitope vaccine construct elevates the levels of antibody titers, cytokine production, B-cell populations, B-cell populations per state, TH-cell populations, and TH-cell populations per state, as shown in **Figure 9**. These findings highlight the potential effectiveness of the multi-epitope vaccine construct in eliciting a comprehensive immune response, demonstrating its ability to induce both humoral and cellular immunity, which is crucial for long-term protection and memory formation. Also, for the experimental validation, the designed vaccine construct has to be produced in higher quantities, and thus usually it will be cloned in a suitable vector (91). In our study, the designed multi-epitope vaccine construct was reversed translated, codon-optimized, and cloned in a suitable vector pET-28a (+) vector-6xHis-TEV-ORF9c (5554 bp) at the restriction sites of SacI (GAGCTC) and EcoRI (GAATTC), as shown in **Figure 10**.

On the other hand, this study mostly used bioinformatics tools and databases for the study, and these computational validations may be less reliable when compared with the experimental validations (92, 93). For instance, the NetCTL 1.2 and NetMHCII 2.3 web servers mainly focus on the limited set of common HLA alleles, potentially overlooking epitopes relevant to underrepresented populations, whereas the VaxiJen v2.0, AllerTOP v.2, ToxinPred, IFNepitope, Expasy ProtParam, and ANTIGENpro web server are commonly used in immunoinformatics and vaccine design approaches; however, these predictions are based on a broad training dataset and do not yield high efficacy as the experimental validations (93, 94). Thus, we strongly recommend to validate the designed vaccine construct in *in vitro* and *in vivo* experimental settings to evaluate their completely therapeutic potential against SLEV. Overall, by employing reverse vaccinology and immunoinformatics approaches, we have designed a multi-epitope cancer vaccine against various key proteins of SLEV such as the membrane glycoprotein M, envelope protein E, and the anchored capsid protein anchC, and we further recommend evaluating its therapeutic potential by *in vitro* and *in vivo* studies in the near future. Furthermore, the deployment of these types of vaccines in regions where diseases are endemic offers significant opportunities to enhance public health and mitigate the disease burden (95). Achieving these outcomes, however, necessitates addressing complex logistical, sociocultural, and economic challenges through well-designed strategies and sustained international cooperation (96). These hurdles could be overcome by strengthening the infrastructure, community engagement, financial support, innovative delivery models, policy and governance, and integrated health programs. Effectively overcoming these barriers is critical to ensuring equitable vaccine access and advancing global objectives in health security and disease control.

## 5 Conclusion

SLEV infection poses a significant public health threat, particularly in regions prone to mosquito-borne diseases. Despite the availability of supportive treatments, there is a critical need for effective therapeutics/vaccines to prevent SLEV infections. In our study, we have designed, constructed, and validated a multi-epitope vaccine targeting key proteins of SLEV such as the membrane glycoprotein M, envelope protein E, and the anchored capsid protein anchC by employing reverse vaccinology and immunoinformatics approaches. Our results indicated that the vaccine construct is structurally stable, antigenic, non−allergic, and non−toxic and has soluble properties. Also, the vaccine exhibited strong binding affinity and structural compactness with the TLR4 upon binding confirmed by docking and molecular dynamics simulations respectively. Furthermore, it also indicated that it has the potential to induce an immune response. Also, it has been cloned in the pET−28a (+) expression vector for the experimental validation by *in vitro* and *in vivo* studies to evaluate the vaccine’s therapeutic efficacy in the near future. Further research and experimental studies are warranted to validate the efficacy, safety, and immunogenicity of the proposed vaccine construct in preclinical and clinical settings.

## Data Availability

The original contributions presented in the study are included in the article/**Supplementary Material**. Further inquiries can be directed to the corresponding author.
