# Supplementary material for: Unveiling reverse vaccinology and immunoinformatics toward Saint Louis encephalitis virus: a ray of hope for vaccine development
Source: Front Immunol. 2025 May 19;16:1576557. doi: 10.3389/fimmu.2025.1576557 (PMC12127316; doi:10.3389/fimmu.2025.1576557)
Supplement: Supplementary file 1 [file DataSheet1.docx]

**Unveiling Reverse Vaccinology and Immunoinformatics Towards Saint Louis Encephalitis Virus: A Ray of Hope for Vaccine Development**

Prasanna Srinivasan Ramalingam^1,†^, Mahalakshmi Aranganathan^1,2,†^, Md Sadique Hussain^3^, Sujatha Elangovan^1^, Gayathri Chellasamy^4^, Purushothaman Balakrishnan^5^, Janaki Ramaiah Mekala^6^, Kyusik Yun^4^, Sivakumar Arumugam^1, *^

^1^Protein Engineering lab, School of Biosciences and Technology, Vellore Institute of Technology, Vellore, TamilNadu, India.

^2^School of Natural Sciences and Mathematics, The University of Texas at Dallas, 800 W. Campbell Road Richardson, Texas, United States.

^3^Uttaranchal Institute of Pharmaceutical Sciences, Uttaranchal University, Dehradun, Uttarakhand 248007, India.

^4^Department of Bionanotechnology, Gachon University, Gyeonggi-do, Republic of Korea.

^5^Department of Biomaterials, Saveetha Dental College and Hospitals, SIMATS, Saveetha University, Chennai-600077, India.

^6^Department of Biotechnology, Koneru Lakshmaiah Education Foundation, Green Fields, Vaddeswaram, Guntur, Andhra Pradesh, India.

^†^These authors contributed equally to this work and share first authorship.

*Corresponding author: [siva_kumar.a@vit.ac.in](mailto:siva_kumar.a@vit.ac.in)

**Supplementary Table 1: Predicted CTL epitopes for Anchored Protein**

| **Peptide** | **Allele** | **VaxiJen Score** | **Allergencity** | **Toxicity** | **ANN 4.0** | **MHC Flurry 2.0** |
| --- | --- | --- | --- | --- | --- | --- |
| GRNRVVNML | B27, B39 | 0.0972 (NON-ANTIGEN) | Non-Allergen | Non-Toxin | ✓ | ✓ |
| RNRVVNMLK | A3 | 0.2975 (NON-ANTIGEN) | Non-Allergen | Non-Toxin | ✓ | ✓ |
| NRVVNMLKR | B27 | 0.2087 (NON-ANTIGEN) | Allergen | Non-Toxin | ✓ | ✓ |
| NMLKRGVSR | A3 | -1.1966 (NON-ANTIGEN) | Allergen | Non-Toxin |  | ✓ |
| MLKRGVSRV | B8 | -0.8086 (NON-ANTIGEN) | Allergen | Non-Toxin |  | ✓ |
| RGVSRVNPL | B7, B8 | 1.1499 (ANTIGEN) | Allergen | Non-Toxin | ✓ | ✓ |
| SRVNPLTGL | B27 | 0.7127 (ANTIGEN) | Non-Allergen | Non-Toxin | ✓ |  |
| RVNPLTGLK | A3 | 1.1321 (ANTIGEN) | Non-Allergen | Non-Toxin | ✓ | ✓ |
| GLKRILGSL | B8 | -1.1538 (NON-ANTIGEN) | Non-Allergen | Non-Toxin |  | ✓ |
| SLLDGRGPV | A2 | 0.0396 (NON-ANTIGEN) | Non-Allergen | Non-Toxin | ✓ | ✓ |
| GRGPVRFIL | B27, B39 | 0.0192 (NON-ANTIGEN) | Allergen | Non-Toxin | ✓ | ✓ |
| GPVRFILAI | B7 | 0.0223 (NON-ANTIGEN) | Allergen | Non-Toxin | ✓ | ✓ |
| RFILAILTF | A24, B58, B62 | 0.0603 (NON-ANTIGEN) | Non-Allergen | Non-Toxin | ✓ | ✓ |
| FILAILTFF | A2, A26, B62 | 0.0040 (NON-ANTIGEN) | Non-Allergen | Non-Toxin | ✓ | ✓ |
| ILAILTFFR | A3 | -0.0642 (NON-ANTIGEN) | Non-Allergen | Non-Toxin | ✓ | ✓ |
| LAILTFFRF | A24, B58, B62 | 0.3346 (NON-ANTIGEN) | Non-Allergen | Non-Toxin | ✓ | ✓ |
| LTFFRFTAL | B8 | 1.0921 (ANTIGEN) | Non-Allergen | Non-Toxin | ✓ | ✓ |
| FRFTALQPT | B27 | 1.5485 (ANTIGEN) | Non-Allergen | Non-Toxin | ✓ | ✓ |
| TALQPTEAL | B7, B39 | 0.7595 (ANTIGEN) | Allergen | Non-Toxin | ✓ | ✓ |
| ALQPTEALK | A3 | 0.8361 (ANTIGEN) | Non-Allergen | Non-Toxin | ✓ | ✓ |
| ALKRRWRAV | B8 | 1.1982 (ANTIGEN) | Non-Allergen | Non-Toxin | ✓ | ✓ |
| KRRWRAVDK | B27 | 1.2236 (ANTIGEN) | Non-Allergen | Non-Toxin | ✓ | ✓ |
| RRWRAVDKR | B27 | 0.5079 (ANTIGEN) | Non-Allergen | Non-Toxin | ✓ | ✓ |
| RAVDKRTAL | B7, B8, B39 | 0.2086 (NON-ANTIGEN) | Allergen | Non-Toxin | ✓ | ✓ |
| AVDKRTALK | A3 | 0.2424 (NON-ANTIGEN) | Allergen | Non-Toxin |  |  |
| TALKHLNGF | A26, B58 | -0.3666 (NON-ANTIGEN) | Non-Allergen | Non-Toxin | ✓ | ✓ |
| ALKHLNGFK | A3 | -0.6153 (NON-ANTIGEN) | Allergen | Non-Toxin |  | ✓ |
| FKRDLGSML | B7 | -0.7853 (NON-ANTIGEN) | Non-Allergen | Non-Toxin | ✓ |  |
| TINRRPSKK | A3 | 0.1348 (NON-ANTIGEN) | Non-Allergen | Non-Toxin | ✓ | ✓ |
| RPSKKRGGT | B7 | -0.7714 (NON-ANTIGEN) | Allergen | Non-Toxin |  | ✓ |
| KKRGGTRSL | B7, B27 | -0.2779 (NON-ANTIGEN) | Allergen | Non-Toxin | ✓ |  |
| KRGGTRSLL | B27, B39 | -0.5535 (NON-ANTIGEN) | Non-Allergen | Non-Toxin | ✓ | ✓ |
| RSLLGLAAL | B62 | 0.5756 (ANTIGEN) | Non-Allergen | Non-Toxin | ✓ |  |
| SLLGLAALI | A2 | 0.6357 (ANTIGEN) | Non-Allergen | Non-Toxin | ✓ | ✓ |

**Note:** The selected epitopes have been shown in highlighted. “✓” denotes predicted.

**Supplementary Table 2: Predicted CTL epitopes for Envelope Protein E**

| **Peptide** | **Allele** | **VaxiJen Score** | **Allergencity** | **Toxicity** | **ANN 4.0** | **MHC Flurry 2.0** |
| --- | --- | --- | --- | --- | --- | --- |
| GATWIDLVL | B58 | 0.9878 (ANTIGEN) | Non-Allergen | Non-Toxin | ✓ | ✓ |
| LEGGSCVTV | B44 | 0.3840 (NON-ANTIGEN) | Allergen | Non-Toxin | ✓ | ✓ |
| VMAPEKPTL | A2, B39, B58, B62 | 0.1958 (NON-ANTIGEN) | Non-Allergen | Non-Toxin | ✓ | ✓ |
| APEKPTLDF | B7 | 0.3814 (NON-ANTIGEN) | Non-Allergen | Non-Toxin | ✓ |  |
| KPTLDFKVM | B7 | 1.0750 (ANTIGEN) | Allergen | Non-Toxin | ✓ |  |
| VMKMEATEL | B8, B62 | 0.6253 (ANTIGEN) | Non-Allergen | Non-Toxin | ✓ | ✓ |
| MEATELATV | B44 | 0.9696 (ANTIGEN) | Non-Allergen | Non-Toxin |  |  |
| TELATVREY | B62 | 0.3897 (NON-ANTIGEN) | Non-Allergen | Non-Toxin |  | ✓ |
| LATVREYCY | B58, B62 | 1.2437 (ANTIGEN) | Non-Allergen | Non-Toxin | ✓ | ✓ |
| REYCYEATL | B39, B44 | 1.0027 (ANTIGEN) | Allergen | Non-Toxin | ✓ | ✓ |
| CYEATLDTL | A24 | 0.2455 (NON-ANTIGEN) | Non-Allergen | Non-Toxin | ✓ |  |
| ATLDTLSTV | A2, A26 | 0.4654 (NON-ANTIGEN) | Non-Allergen | Non-Toxin | ✓ | ✓ |
| NTKRSDPTF | B62 | 1.6379 (ANTIGEN) | Allergen | Non-Toxin | ✓ |  |
| RSDPTFVCK | A3 | 1.9623 (ANTIGEN) | Non-Allergen | Non-Toxin | ✓ | ✓ |
| GWGNGCGLF | A24 | -0.7923 (NON-ANTIGEN) | Non-Allergen | Non-Toxin | ✓ |  |
| GSIDTCAKF | A26, B58, B62 | -0.3220 (NON-ANTIGEN) | Allergen | Non-Toxin | ✓ | ✓ |
| NKATGKTIL | B39 | -0.1831 (NON-ANTIGEN) | Non-Allergen | Non-Toxin | ✓ | ✓ |
| KATGKTILR | A3 | 0.1311 (NON-ANTIGEN) | Allergen | Non-Toxin | ✓ | ✓ |
| KTILRENIK | A3 | -0.3351 (NON-ANTIGEN) | Non-Allergen | Non-Toxin | ✓ | ✓ |
| TILRENIKY | A1, A3, A26 | 0.0648 (NON-ANTIGEN) | Allergen | Non-Toxin | ✓ | ✓ |
| NIKYEVAIF | A26, B8 | 0.7231 (ANTIGEN) | Non-Allergen | Non-Toxin | ✓ | ✓ |
| IKYEVAIFV | A2 | 0.2126 (NON-ANTIGEN) | Non-Allergen | Non-Toxin | ✓ | ✓ |
| TDSTSHGNY | A1 | 0.4931 (NON-ANTIGEN) | Non-Allergen | Non-Toxin |  | ✓ |
| ARFTISPQA | B27 | 1.3185 (ANTIGEN) | Allergen | Non-Toxin |  | ✓ |
| TISPQAPSF | B58, B62 | 0.9882 (ANTIGEN) | Non-Allergen | Non-Toxin | ✓ | ✓ |
| SPQAPSFTA | B7 | 0.7395 (ANTIGEN) | Non-Allergen | Non-Toxin | ✓ | ✓ |
| SFTANMGEY | A1, A26, B62 | 0.3571 (NON-ANTIGEN) | Non-Allergen | Non-Toxin | ✓ | ✓ |
| RSGINTEDY | A1, B53, B62 | 0.7970 (ANTIGEN) | Non-Allergen | Non-Toxin | ✓ | ✓ |
| SGINTEDYY | A26 | 1.0251 (ANTIGEN) | Allergen | Non-Toxin | ✓ | ✓ |
| GINTEDYYV | A2 | 0.9885 (ANTIGEN) | Allergen | Non-Toxin |  |  |
| INTEDYYVF | B39 | 0.5378 (ANTIGEN) | Allergen | Non-Toxin | ✓ | ✓ |
| NTEDYYVFT | A1 | 0.3099 (NON-ANTIGEN) | Allergen | Non-Toxin | ✓ | ✓ |
| TEDYYVFTV | B44 | 0.4447 (NON-ANTIGEN) | Non-Allergen | Non-Toxin | ✓ | ✓ |
| YYVFTVKEK | A24 | 1.6558 (ANTIGEN) | Non-Allergen | Non-Toxin |  | ✓ |
| KSWLVNRDW | B58 | 0.2463 (NON-ANTIGEN) | Allergen | Non-Toxin | ✓ | ✓ |
| SWLVNRDWF | A24 | 0.2678 (NON-ANTIGEN) | Allergen | Non-Toxin |  | ✓ |
| VNRDWFHDL | B39 | 0.0361 (NON-ANTIGEN) | Allergen | Non-Toxin | ✓ |  |
| RDWFHDLNL | B44 | 0.5836 (ANTIGEN) | Allergen | Non-Toxin |  | ✓ |
| LPWTSPATT | B7 | 0.6743 (ANTIGEN) | Allergen | Non-Toxin | ✓ | ✓ |
| WTSPATTDW | A1, B58 | 0.7328 (ANTIGEN) | Allergen | Non-Toxin | ✓ | ✓ |
| RNRETLVEF | B62 | 0.8290 (ANTIGEN) | Non-Allergen | Non-Toxin | ✓ |  |
| EPHATKQTV | B8 | 0.1990 (NON-ANTIGEN) | Non-Allergen | Non-Toxin | ✓ |  |
| ATKQTVVAL | B7, B8, B62 | 0.8144 (ANTIGEN) | Non-Allergen | Non-Toxin | ✓ | ✓ |
| QEGALHTAL | B44 | 0.0421 (NON-ANTIGEN) | Allergen | Non-Toxin | ✓ | ✓ |
| ALHTALAGA | A2 | 0.1904 (NON-ANTIGEN) | Non-Allergen | Non-Toxin | ✓ | ✓ |
| ALAGAIPAT | A2 | 0.3726 (NON-ANTIGEN) | Non-Allergen | Non-Toxin |  | ✓ |
| IPATVSSST | B7 | 0.4426 (NON-ANTIGEN) | Allergen | Non-Toxin | ✓ | ✓ |
| TVSSSTLTL | B7, B39 | 0.4616 (NON-ANTIGEN) | Allergen | Non-Toxin | ✓ | ✓ |
| LTLQSGHLK | A3 | 1.1646 (ANTIGEN) | Non-Allergen | Non-Toxin | ✓ | ✓ |
| GHLKCRAKL | B39 | 1.6030 (ANTIGEN) | Allergen | Non-Toxin | ✓ | ✓ |
| KVKIKGTTY | A3, B8, B62 | 1.1085 (ANTIGEN) | Allergen | Non-Toxin | ✓ | ✓ |
| KIKGTTYGM | A26, B8 | 0.8519 (ANTIGEN) | Allergen | Non-Toxin | ✓ | ✓ |
| TYGMCDSAF | A24 | -0.1242 (NON-ANTIGEN) | Non-Allergen | Non-Toxin | ✓ | ✓ |
| GMCDSAFTF | B58, B62 | 0.3707 (NON-ANTIGEN) | Non-Allergen | Non-Toxin | ✓ | ✓ |
| PTDTGHGTV | A1 | 1.0702 (ANTIGEN) | Allergen | Non-Toxin |  | ✓ |
| GHGTVIVEL | B39 | 0.2531 (NON-ANTIGEN) | Non-Allergen | Non-Toxin | ✓ | ✓ |
| GTVIVELQY | A1, B58 | 1.3324 (ANTIGEN) | Allergen | Non-Toxin | ✓ | ✓ |
| GPCRVPISV | B7 | -0.3434 (NON-ANTIGEN) | Allergen | Non-Toxin | ✓ | ✓ |
| VPISVTANL | B7 | 1.0379 (ANTIGEN) | Non-Allergen | Non-Toxin | ✓ | ✓ |
| TPVGRLVTV | B7 | 0.1831 (NON-ANTIGEN) | Non-Allergen | Non-Toxin | ✓ | ✓ |
| GRLVTVNPF | B27 | 0.2528 (NON-ANTIGEN) | Allergen | Non-Toxin |  |  |
| RLVTVNPFI | A2 | 0.1149 (NON-ANTIGEN) | Allergen | Non-Toxin | ✓ | ✓ |
| ISTGGANNK | A3 | 1.3662 (ANTIGEN) | Allergen | Non-Toxin |  | ✓ |
| VMIEVEPPF | A24, B58, B62 | 1.1906 (ANTIGEN) | Allergen | Non-Toxin | ✓ | ✓ |
| GRGTTQINY | B27 | 1.4582 (ANTIGEN) | Allergen | Non-Toxin | ✓ | ✓ |
| GTTQINYHW | B58 | 1.7161 (ANTIGEN) | Allergen | Non-Toxin | ✓ | ✓ |
| TQINYHWHK | A3 | 0.8026 (ANTIGEN) | Allergen | Non-Toxin | ✓ | ✓ |
| HWHKEGSSI | A24 | 0.5558 (ANTIGEN) | Non-Allergen | Non-Toxin | ✓ | ✓ |
| HKEGSSIGK | B27 | 0.7236 (ANTIGEN) | Allergen | Non-Toxin | ✓ |  |
| EGSSIGKAL | B8 | 0.2926 (NON-ANTIGEN) | Allergen | Non-Toxin |  | ✓ |
| IGKALATTW | B58 | -0.0409 (NON-ANTIGEN) | Allergen | Non-Toxin | ✓ | ✓ |
| KGAQRLAVL | B8 | 0.2840 (NON-ANTIGEN) | Non-Allergen | Non-Toxin | ✓ | ✓ |
| LAVLGDTAW | B58 | 0.7980 (ANTIGEN) | Allergen | Non-Toxin | ✓ | ✓ |
| VLGDTAWDF | B62 | 1.5121 (ANTIGEN) | Allergen | Non-Toxin | ✓ | ✓ |
| DTAWDFGSI | A26 | 1.8803 (ANTIGEN) | Non-Allergen | Non-Toxin | ✓ | ✓ |
| DFGSIGGVF | A24 | 1.1445 (ANTIGEN) | Allergen | Non-Toxin | ✓ | ✓ |
| SIGKAVHQV | A2 | 0.4724 (NON-ANTIGEN) | Non-Allergen | Non-Toxin | ✓ | ✓ |
| IGKAVHQVF | B8, B58, B62 | 0.3277 (NON-ANTIGEN) | Non-Allergen | Non-Toxin | ✓ | ✓ |
| VHQVFGGAF | B8 | -0.0581 (NON-ANTIGEN) | Non-Allergen | Non-Toxin | ✓ | ✓ |
| VFGGAFRTL | A24 | -0.2548 (NON-ANTIGEN) | Allergen | Non-Toxin | ✓ | ✓ |
| RTLFGGMSW | B58, B62 | 0.6566 (ANTIGEN) | Allergen | Non-Toxin | ✓ | ✓ |
| TLFGGMSWI | A2, A26 | 0.3054 (NON-ANTIGEN) | Non-Allergen | Non-Toxin | ✓ | ✓ |
| GMSWITQGL | A2, B62 | 0.8706 (ANTIGEN) | Non-Allergen | Non-Toxin | ✓ | ✓ |
| GLLGALLLW | B58 | 0.2073 (NON-ANTIGEN) | Non-Allergen | Non-Toxin |  |  |
| LLGALLLWM | A2 | -0.0171 (NON-ANTIGEN) | Non-Allergen | Non-Toxin |  | ✓ |
| LLLWMGLQA | A2 | 0.6261 (ANTIGEN) | Allergen | Non-Toxin |  | ✓ |
| LLWMGLQAR | A3 | 1.7758 (ANTIGEN) | Allergen | Non-Toxin | ✓ | ✓ |
| QARDRSISL | B7, B8, B62 | 2.1371 (ANTIGEN) | Allergen | Non-Toxin |  | ✓ |
| RDRSISLTL | B7, B8, B44 | 2.1072 (ANTIGEN) | Non-Allergen | Non-Toxin | ✓ | ✓ |
| SISLTLLAV | A2 | 1.4839 (ANTIGEN) | Non-Allergen | Non-Toxin |  |  |
| LLAVGGILI | A2 | 0.5101 (ANTIGEN) | Non-Allergen | Non-Toxin | ✓ | ✓ |
| LAVGGILIF | B58, B62 | 0.4342 (NON-ANTIGEN) | Non-Allergen | Non-Toxin | ✓ | ✓ |
| AVGGILIFL | A2 | 0.0773 (NON-ANTIGEN) | Allergen | Non-Toxin | ✓ | ✓ |
| ILIFLATSV | A2 | 0.3672 (NON-ANTIGEN) | Non-Allergen | Non-Toxin | ✓ | ✓ |

**Note:** The selected epitopes have been shown in highlighted. “✓” denotes predicted.

**Supplementary Table 3: Predicted CTL epitopes for Membrane Protein**

| **Peptide** | **Allele** | **VaxiJen Score** | **Allergencity** | **Toxicity** | **ANN 4.0** | **MHC Flurry 2.0** |
| --- | --- | --- | --- | --- | --- | --- |
| VQHHGDSTL | B39, B44, B62 | 0.0180 (NON-ANTIGEN) | Allergen | Non-Toxin | ✓ | ✓ |
| HHGDSTLAT | B39 | 0.4090 (NON-ANTIGEN) | Allergen | Non-Toxin | ✓ | ✓ |
| TLATKNTPW | B58 | 1.6663 (ANTIGEN) | Non-Allergen | Non-Toxin | ✓ | ✓ |
| WLDTVKTTK | A1 | 0.5272 (ANTIGEN) | Allergen | Non-Toxin | ✓ | ✓ |
| DTVKTTKYL | A26 | 0.3000 (NON-ANTIGEN) | Non-Allergen | Non-Toxin |  | ✓ |
| KTTKYLTKV | A2 | 0.2300 (NON-ANTIGEN) | Non-Allergen | Non-Toxin | ✓ |  |
| KYLTKVENW | A24, B58 | 0.3090 (NON-ANTIGEN) | Non-Allergen | Non-Toxin | ✓ | ✓ |
| YLTKVENWV | A2 | 0.2890 (NON-ANTIGEN) | Non-Allergen | Non-Toxin | ✓ | ✓ |
| LTKVENWVL | B8 | 0.4350 (NON-ANTIGEN) | Non-Allergen | Non-Toxin | ✓ | ✓ |
| VLRNPGYAL | B7, B8, B62 | -0.1984 (NON-ANTIGEN) | Allergen | Non-Toxin | ✓ | ✓ |
| NPGYALVAL | B7 | 0.9540 (ANTIGEN) | Non-Allergen | Non-Toxin | ✓ |  |
| GYALVALAI | A24 | 0.8890 (ANTIGEN) | Non-Allergen | Non-Toxin |  |  |
| ALVALAIGW | B58 | 1.6221 (ANTIGEN) | Non-Allergen | Non-Toxin | ✓ |  |
| VALAIGWML | B58 | 1.3935 (ANTIGEN) | Non-Allergen | Non-Toxin |  | ✓ |
| MLGSNNTQR | A3 | 0.5967 (ANTIGEN) | Non-Allergen | Non-Toxin | ✓ |  |
| NTQRVVFVI | A24 | 0.7472 (ANTIGEN) | Non-Allergen | Non-Toxin | ✓ | ✓ |
| TQRVVFVIM | B62 | 0.8083 (ANTIGEN) | Non-Allergen | Non-Toxin |  | ✓ |
| QRVVFVIML | B37, B39 | 0.7615 (ANTIGEN) | Non-Allergen | Non-Toxin | ✓ | ✓ |
| RVVFVIMLM | A3, A26 | 0.7481 (ANTIGEN) | Non-Allergen | Non-Toxin | ✓ | ✓ |
| VVFVIMLML | A2 | 0.5464 (ANTIGEN) | Non-Allergen | Non-Toxin |  | ✓ |
| VFVIMLMLI | A24 | 0.2550 (NON-ANTIGEN) | Non-Allergen | Non-Toxin |  |  |
| IMLMLIAPA | A2 | 0.3164 (NON-ANTIGEN) | Non-Allergen | Non-Toxin | ✓ | ✓ |
| MLMLIAPAY | A1, A3, B62 | 0.4413 (NON-ANTIGEN) | Allergen | Non-Toxin | ✓ | ✓ |

**Note:** The selected epitopes have been shown in highlighted. “✓” denotes predicted.

**Supplementary Table 4: Predicted HTL epitopes against Membrane Glycoprotein**

| **Peptide** | **Alleles** | **VaxiJen** | **Allergencity** | **Toxicity** | **IFN-γ** | **Comblib** | **Tepitope** |
| --- | --- | --- | --- | --- | --- | --- | --- |
| AIGWMLGSNNTQRVV | DRB1_0401, DRB1_0402, DRB1_1301, DRB1_1302, DRB3_0202, DRB3_0301 | 0.5290 (ANTIGEN) | Allergen | Non-Toxin | Positive |  | ✓ |
| ALAIGWMLGSNNTQR | DRB1_0402, DRB1_0403, DRB3_0202, HLA-DQA10104-DQB10503 | 0.8390 (ANTIGEN) | Non-Allergen | Non-Toxin | Positive | ✓ | ✓ |
| ALVALAIGWMLGSNN | DRB1_0402, DRB1_0403, DRB1_0701, HLA-DQA10101-DQB10501, HLA-DQA10104-DQB10503, HLA-DQA10301-DQB10301 | 0.8685 (ANTIGEN) | Non-Allergen | Non-Toxin | Negative | ✓ | ✓ |
| GWMLGSNNTQRVVFV | DRB1_1301, DRB1_1302, DRB3_0202, DRB3_0301 | 0.5186 (ANTIGEN) | Allergen | Non-Toxin | Positive | ✓ | ✓ |
| GYALVALAIGWMLGS | DRB1_0402, DRB1_0403, DRB1_0701, DRB1_1001, HLA-DQA10301-DQB10301, HLA-DQA10501-DQB10303 | 0.9121 (ANTIGEN) | Non-Allergen | Non-Toxin | Negative | ✓ | ✓ |
| IGWMLGSNNTQRVVF | DRB1_0401, DRB1_1301, DRB1_1302, DRB3_0202, DRB3_0301 | 0.6726 (ANTIGEN) | Allergen | Non-Toxin | Positive | ✓ | ✓ |
| LAIGWMLGSNNTQRV | DRB1_0401, DRB1_0402, DRB1_1302, DRB3_0202, DRB3_0301, HLA-DQA10104-DQB10503 | 0.8559 (ANTIGEN) | Allergen | Non-Toxin | Positive | ✓ |  |
| LRNPGYALVALAIGW | HLA-DQA10103-DQB10603, HLA-DQA10301-DQB10301, HLA-DQA10303-DQB10402, HLA-DQA10501-DQB10301, HLA-DQA10501-DQB10302, HLA-DQA10501-DQB10303, HLA-DQA10501-DQB10402 | 0.9822 (ANTIGEN) | Non-Allergen | Non-Toxin | Negative | ✓ |  |
| LVALAIGWMLGSNNT | DRB1_0402, DRB1_0403, HLA-DQA10101-DQB10501, HLA-DQA10104-DQB10503, HLA-DQA10301-DQB10301 | 0.8573 (ANTIGEN) | Non-Allergen | Non-Toxin | Negative | ✓ |  |
| MLGSNNTQRVVFVIM | DRB3_0202, DRB3_0301 | 0.6753 (ANTIGEN) | Allergen | Non-Toxin | Negative |  |  |
| NPGYALVALAIGWML | DRB1_0101, DRB1_0402, DRB1_0403, DRB1_0701, DRB1_0901, DRB1_1001, HLA-DQA10301-DQB10301, HLA-DQA10303-DQB10402, HLA-DQA10501-DQB10301, HLA-DQA10501-DQB10302, HLA-DQA10501-DQB10303, HLA-DQA10501-DQB10402 | 1.1365 (ANTIGEN) | Non-Allergen | Non-Toxin | Negative | ✓ | ✓ |
| NTQRVVFVIMLMLIA | DRB1_0103, HLA-DPA10103-DPB10601 | 0.5387 (ANTIGEN) | Non-Allergen | Non-Toxin | Negative | ✓ | ✓ |
| NWVLRNPGYALVALA | DRB1_0101, DRB1_0701, DRB1_1302, DRB1_1501, DRB3_0202, DRB3_0301, DRB4_0103, HLA-DPA10103-DPB10301, HLA-DQA10301-DQB10301, HLA-DQA10303-DQB10402, HLA-DQA10501-DQB10301, HLA-DQA10501-DQB10303 | 0.5813 (ANTIGEN) | Allergen | Non-Toxin | Negative | ✓ |  |
| PGYALVALAIGWMLG | DRB1_0101, DRB1_0402, DRB1_0403, DRB1_0701, DRB1_0901, DRB1_1001, HLA-DQA10301-DQB10301, HLA-DQA10501-DQB10301, HLA-DQA10501-DQB10303 | 0.8564 (ANTIGEN) | Non-Allergen | Non-Toxin | Negative |  |  |
| QRVVFVIMLMLIAPA | DRB1_0103, DRB1_0403, HLA-DPA10103-DPB10601, HLA-DQA10102-DQB10501 | 0.5393 (ANTIGEN) | Non-Allergen | Non-Toxin | Negative |  | ✓ |
| RNPGYALVALAIGWM | DRB1_0402, DRB1_0403, HLA-DQA10301-DQB10301, HLA-DQA10303-DQB10402, HLA-DQA10501-DQB10301, HLA-DQA10501-DQB10302, HLA-DQA10501-DQB10303, HLA-DQA10501-DQB10402 | 1.1743 (ANTIGEN) | Non-Allergen | Non-Toxin | Negative |  | ✓ |
| RVVFVIMLMLIAPAY | DRB1_0103, DRB1_0403, DRB1_0802, DRB1_0901, DRB1_1201, HLA-DPA10103-DPB10601, HLA-DQA10102-DQB10501 | 0.5751 (ANTIGEN) | Non-Allergen | Non-Toxin | Negative |  | ✓ |
| SNNTQRVVFVIMLML | DRB1_0103, HLA-DPA10103-DPB10601 | 0.5165 (ANTIGEN) | Non-Allergen | Non-Toxin | Negative | ✓ | ✓ |
| TQRVVFVIMLMLIAP | DRB1_0103, HLA-DPA10103-DPB10601 | 0.5350 (ANTIGEN) | Non-Allergen | Non-Toxin | Negative | ✓ | ✓ |
| VALAIGWMLGSNNTQ | DRB1_0402, DRB1_0403, HLA-DQA10101-DQB10501, HLA-DQA10104-DQB10503 | 0.9820 (ANTIGEN) | Non-Allergen | Non-Toxin | Negative |  | ✓ |
| VLRNPGYALVALAIG | DRB1_1302, HLA-DPA10103-DPB10301, HLA-DPA10201-DPB11401, HLA-DQA10301-DQB10301, HLA-DQA10303-DQB10402, HLA-DQA10501-DQB10301, HLA-DQA10501-DQB10302, HLA-DQA10501-DQB10303 | 0.5479 (ANTIGEN) | Allergen | Non-Toxin | Negative | ✓ |  |
| WMLGSNNTQRVVFVI | DRB1_1301, DRB1_1302, DRB3_0202, DRB3_0301 | 0.6457 (ANTIGEN) | Allergen | Non-Toxin | Positive | ✓ | ✓ |
| WVLRNPGYALVALAI | DRB1_0101, DRB1_0701, DRB1_0901, DRB1_1302, DRB1_1501, DRB3_0202, DRB3_0301, HLA-DPA10103-DPB10301, HLA-DPA10201-DPB11401, HLA-DQA10301-DQB10301, HLA-DQA10303-DQB10402, HLA-DQA10501-DQB10301, HLA-DQA10501-DQB10302, HLA-DQA10501-DQB10303, HLA-DQA10501-DQB10402 | 0.5629 (ANTIGEN) | Allergen | Non-Toxin | Negative | ✓ |  |
| YALVALAIGWMLGSN | DRB1_0402, DRB1_0403, DRB1_0701, HLA-DQA10301-DQB10301 | 0.8859 (ANTIGEN) | Non-Allergen | Non-Toxin | Negative |  | ✓ |

**Note:** The selected epitopes have been shown in highlighted. “✓” denotes predicted.

**Supplementary Table 5: Predicted HTL epitopes against Envelope Protein**

| **Peptide** | **Alleles** | **VaxiJen** | **Allergencity** | **Toxicity** | **IFN-γ** | **Comblib** | **Tepitope** |
| --- | --- | --- | --- | --- | --- | --- | --- |
| DFGSIGGVFNSIGKA | HLA-DQA10301-DQB10301, HLA-DQA10501-DQB10301, HLA-DQA10501-DQB10302 | 0.6480 (ANTIGEN) | NON-ALLERGEN | Non-Toxin | POSITIVE | ✓ | ✓ |
| EKPTLDFKVMKMEAT | DRB1_0103, DRB1_0801, DRB1_1001, DRB1_1101, DRB4_0101, HLA-DQA10201-DQB10402 | 0.7409 (ANTIGEN) | NON-ALLERGEN | Non-Toxin | POSITIVE | ✓ | ✓ |
| GALLLWMGLQARDRS | DRB1_0402, DRB1_1301, DRB1_1501, DRB1_1602, DRB4_0101, DRB4_0103, DRB5_0101, HLA-DQA10102-DQB10502, HLA-DQA10201-DQB10402, HLA-DQA10303-DQB10402, HLA-DQA10501-DQB10402, HLA-DQA10601-DQB10402 | 1.3932 (ANTIGEN) | NON-ALLERGEN | Non-Toxin | POSITIVE |  | ✓ |
| GASGATWIDLVLEGG | HLA-DPA10103-DPB10402, HLA-DQA10101-DQB10501, HLA-DQA10301-DQB10302, HLA-DQA10401-DQB10402, HLA-DQA10501-DQB10201 | 0.6781 (ANTIGEN) | NON-ALLERGEN | Non-Toxin | POSITIVE | ✓ | ✓ |
| GLLGALLLWMGLQAR | DRB1_0402, DRB1_1501, DRB4_0101, DRB4_0103, HLA-DPA10103-DPB10402, HLA-DQA10303-DQB10402 | 0.9933 (ANTIGEN) | NON-ALLERGEN | Non-Toxin | POSITIVE | ✓ |  |
| HATKQTVVALGSQEG | HLA-DPA10201-DPB11401, HLA-DQA10103-DQB10603, HLA-DQA10201-DQB10301, HLA-DQA10201-DQB10303, HLA-DQA10501-DQB10303 | 0.7508 (ANTIGEN) | NON-ALLERGEN | Non-Toxin | POSITIVE | ✓ |  |
| KMEATELATVREYCY | HLA-DQA10102-DQB10602, HLA-DQA10201-DQB10202, HLA-DQA10301-DQB10302, HLA-DQA10401-DQB10402 | 0.9318 (ANTIGEN) | NON-ALLERGEN | Non-Toxin | POSITIVE | ✓ | ✓ |
| KQTVVALGSQEGALH | DRB1_0101, HLA-DQA10103-DQB10603, HLA-DQA10201-DQB10303, HLA-DQA10501-DQB10303 | 0.7905 (ANTIGEN) | NON-ALLERGEN | Non-Toxin | POSITIVE |  |  |
| LFGGMSWITQGLLGA | DRB1_0402, HLA-DQA10103-DQB10603, HLA-DQA10201-DQB10402, HLA-DQA10501-DQB10301, HLA-DQA10501-DQB10302 | 0.7270 (ANTIGEN) | NON-ALLERGEN | Non-Toxin | POSITIVE | ✓ | ✓ |
| LGALLLWMGLQARDR | DRB1_0402, DRB1_1301, DRB1_1501, DRB1_1602, DRB4_0101, DRB4_0103, DRB5_0101, HLA-DQA10102-DQB10502, HLA-DQA10303-DQB10402, HLA-DQA10501-DQB10402, HLA-DQA10601-DQB10402 | 1.4396 (ANTIGEN) | NON-ALLERGEN | Non-Toxin | POSITIVE | ✓ | ✓ |
| LLGALLLWMGLQARD | DRB1_0402, DRB1_1501, DRB4_0101, DRB4_0103, HLA-DQA10303-DQB10402 | 1.0866 (ANTIGEN) | NON-ALLERGEN | Non-Toxin | POSITIVE | ✓ |  |
| LNLPWTSPATTDWRN | HLA-DQA10201-DQB10303, HLA-DQA10201-DQB10402, HLA-DQA10501-DQB10302, HLA-DQA10601-DQB10402 | 1.2603 (ANTIGEN) | NON-ALLERGEN | Non-Toxin | POSITIVE |  | ✓ |
| LVTVNPFISTGGANN | DRB1_1001, HLA-DQA10102-DQB10501, HLA-DQA10103-DQB10603, HLA-DQA10201-DQB10301, HLA-DQA10201-DQB10303, HLA-DQA10201-DQB10402, HLA-DQA10501-DQB10303 | 0.5279 (ANTIGEN) | NON-ALLERGEN | Non-Toxin | POSITIVE | ✓ | ✓ |
| MKMEATELATVREYC | HLA-DQA10102-DQB10602, HLA-DQA10201-DQB10202, HLA-DQA10301-DQB10302, HLA-DQA10401-DQB10402 | 0.8154 (ANTIGEN) | NON-ALLERGEN | Non-Toxin | POSITIVE | ✓ | ✓ |
| MSWITQGLLGALLLW | DRB1_0402, DRB1_1201, HLA-DPA10103-DPB10201, HLA-DQA10201-DQB10301, HLA-DQA10201-DQB10303 | 0.5673 (ANTIGEN) | NON-ALLERGEN | Non-Toxin | POSITIVE | ✓ | ✓ |
| NLPWTSPATTDWRNR | HLA-DQA10201-DQB10402, HLA-DQA10303-DQB10402, HLA-DQA10501-DQB10302, HLA-DQA10601-DQB10402 | 1.4470 (ANTIGEN) | NON-ALLERGEN | Non-Toxin | POSITIVE | ✓ | ✓ |
| PQAPSFTANMGEYGT | DRB3_0202, HLA-DQA10103-DQB10603, HLA-DQA10501-DQB10302 | 0.6128 (ANTIGEN) | NON-ALLERGEN | Non-Toxin | POSITIVE | ✓ | ✓ |
| PTLDFKVMKMEATEL | DRB1_0101, DRB1_0103, DRB1_0401, DRB1_0405, DRB1_0701, DRB1_0801, DRB1_0901, DRB1_1001, DRB1_1101, DRB1_1201, DRB1_1602, DRB4_0101, HLA-DPA10103-DPB10402, HLA-DPA10103-DPB10601, HLA-DQA10201-DQB10402, HLA-DQA10501-DQB10201 | 0.9687 (ANTIGEN) | NON-ALLERGEN | Non-Toxin | POSITIVE | ✓ | ✓ |
| REYCYEATLDTLSTV | DRB1_0401, DRB1_0405, HLA-DPA10103-DPB10601, HLA-DPA10103-DPB10201, HLA-DQA10201-DQB10202 | 0.6329 (ANTIGEN) | NON-ALLERGEN | Non-Toxin | POSITIVE |  | ✓ |
| SGINTEDYYVFTVKE | HLA-DPA10103-DPB10201, HLA-DQA10104-DQB10503, HLA-DQA10301-DQB10302, HLA-DQA10401-DQB10402 | 0.8979 (ANTIGEN) | NON-ALLERGEN | Non-Toxin | POSITIVE | ✓ | ✓ |
| TIDCEARSGINTEDY | HLA-DQA10301-DQB10302, HLA-DQA10401-DQB10402 | 0.9033 (ANTIGEN) | NON-ALLERGEN | Non-Toxin | POSITIVE | ✓ | ✓ |
| TKQTVVALGSQEGAL | DRB1_0101, HLA-DQA10103-DQB10603, HLA-DQA10201-DQB10301, HLA-DQA10201-DQB10303, HLA-DQA10501-DQB10303 | 0.8495 (ANTIGEN) | NON-ALLERGEN | Non-Toxin | POSITIVE | ✓ | ✓ |
| TTQINYHWHKEGSSI | HLA-DQA10104-DQB10503, HLA-DQA10601-DQB10402 | 0.7808 (ANTIGEN) | NON-ALLERGEN | Non-Toxin | POSITIVE |  | ✓ |
| TVREYCYEATLDTLS | DRB1_0401, DRB1_0405, HLA-DPA10103-DPB10601, HLA-DPA10103-DPB10201, HLA-DQA10201-DQB10202, HLA-DQA10501-DQB10201 | 0.5897 (ANTIGEN) | NON-ALLERGEN | Non-Toxin | POSITIVE |  | ✓ |

**Note:** The selected epitopes have been shown in highlighted. “✓” denotes predicted.

**Supplementary Table 6: Predicted HTL epitopes against Anchored Protein**

| **Peptide** | **Alleles** | **VaxiJen** | **Allergencity** | **Toxicity** | **IFN-γ** | **Comblib** | **Tepitope** |
| --- | --- | --- | --- | --- | --- | --- | --- |
| ALKRRWRAVDKRTAL | DRB1_0801, DRB1_1101, DRB1_1301, DRB4_0103, DRB5_0101, HLA-DPA10103-DPB10301, HLA-DQA1003-DQB10402 | 0.9121 (ANTIGEN) | NON-ALLERGEN | Non-Toxin | POSITIVE | ✓ | ✓ |
| ALQPTEALKRRWRAV | DRB1_1301, DRB4_0103 | 0.8195 (ANTIGEN) | NON-ALLERGEN | Non-Toxin | POSITIVE | ✓ |  |
| EALKRRWRAVDKRTA | DRB1_0801, DRB1_1101, DRB1_1301, DRB4_0103, DRB5_0101, HLA-DPA10103-DPB10301, HLA-DQA10303-DQB10402 | 0.6282 (ANTIGEN) | NON-ALLERGEN | Non-Toxin | POSITIVE | ✓ | ✓ |
| FTALQPTEALKRRWR | DRB1_0101, DRB1_0901, DRB1_1001, DRB3_0202, DRB5_0101 | 0.7429 (ANTIGEN) | NON-ALLERGEN | Non-Toxin | POSITIVE |  | ✓ |
| ILTFFRFTALQPTEA | DRB1_0101, DRB1_0401, DRB1_0402, DRB1_0404, DRB1_0405, DRB1_0701, DRB1_0801, DRB1_0802, DRB1_0901, DRB1_1001, DRB1_1101, DRB1_1602, DRB4_0103, HLA-DPA10103-DPB10301, HLA-DPA10103-DPB10401, HLA-DPA10103-DPB10601, HLA-DPA10201-DPB10101, HLA-DPA10201-DPB10501, HLA-DPA10201-DPB11401, HLA-DPA10301-DPB10402, HLA-DPA10103-DPB10201, HLA-DQA10101-DQB10501, HLA-DQA10102-DQB10602, HLA-DQA10104-DQB10503, HLA-DQA10201-DQB10202, HLA-DQA10201-DQB10402, HLA-DQA10301-DQB10302, HLA-DQA10401-DQB10402, HLA-DQA10501-DQB10201, HLA-DQA10501-DQB10302, HLA-DQA10501-DQB10402, HLA-DQA10601-DQB10402 | 0.7132 (ANTIGEN) | NON-ALLERGEN | Non-Toxin | POSITIVE | ✓ | ✓ |
| KRRWRAVDKRTALKH | DRB1_0301, DRB1_0801, DRB1_1101, DRB1_1602, DRB4_0103, DRB5_0101, HLA-DPA10103-DPB10301, HLA-DQA10303-DQB10402 | 0.5483 (ANTIGEN) | NON-ALLERGEN | Non-Toxin | POSITIVE | ✓ | ✓ |
| LKRRWRAVDKRTALK | DRB1_0301, DRB1_0801, DRB1_1101, DRB1_1301, DRB4_0103, DRB5_0101, HLA-DPA10103-DPB10301, HLA-DQA10303-DQB10402 | 0.6857 (ANTIGEN) | NON-ALLERGEN | Non-Toxin | POSITIVE | ✓ | ✓ |
| LQPTEALKRRWRAVD | DRB1_0801, DRB1_1301, DRB4_0103 | 1.1347 (ANTIGEN) | NON-ALLERGEN | Non-Toxin | POSITIVE |  |  |
| PTEALKRRWRAVDKR | DRB1_0801, DRB1_1101, DRB1_1301, DRB4_0103, DRB5_0101, HLA-DPA10103-DPB10301, HLA-DQA10303-DQB10402 | 0.6316 (ANTIGEN) | NON-ALLERGEN | Non-Toxin | POSITIVE | ✓ |  |
| QPTEALKRRWRAVDK | DRB1_0801, DRB1_1301, DRB4_0103 | 0.6643 (ANTIGEN) | NON-ALLERGEN | Non-Toxin | POSITIVE |  | ✓ |
| TALQPTEALKRRWRA | DRB1_1301, DRB4_0103 | 0.6880 (ANTIGEN) | NON-ALLERGEN | Non-Toxin | POSITIVE |  | ✓ |
| TEALKRRWRAVDKRT | DRB1_0801, DRB1_1101, DRB1_1301, DRB4_0103, DRB5_0101, HLA-DPA10103-DPB10301, HLA-DQA10303-DQB10402 | 0.5894 (ANTIGEN) | NON-ALLERGEN | Non-Toxin | POSITIVE | ✓ | ✓ |

**Note:** The selected epitopes have been shown in highlighted. “✓” denotes predicted.

**Supplementary Table 7: Selected CTL epitopes of all target proteins**

| **Peptide** | **Allele** | **VaxiJen Score** | **Allergencity** | **Toxicity** |
| --- | --- | --- | --- | --- |
| NIKYEVAIF | A26, B8 | 0.7231 (ANTIGEN) | Non-Allergen | Non-Toxin |
| TISPQAPSF | B58, B62 | 0.9882 (ANTIGEN) | Non-Allergen | Non-Toxin |
| RDRSISLTL | B7, B8, B44 | 2.1072 (ANTIGEN) | Non-Allergen | Non-Toxin |
| QRVVFVIML | B37, B39 | 0.7615 (ANTIGEN) | Non-Allergen | Non-Toxin |
| RVVFVIMLM | A3, A26 | 0.7481 (ANTIGEN) | Non-Allergen | Non-Toxin |

**Supplementary Table 8: Selected HTL epitopes of all target proteins**

| **Peptide** | **Alleles** | **VaxiJen** | **Allergencity** | **Toxicity** | **IFN** |
| --- | --- | --- | --- | --- | --- |
| ALAIGWMLGSNNTQR | DRB1_0402, DRB1_0403, DRB3_0202, HLA-DQA10104-DQB10503 | 0.8390 (ANTIGEN) | Non-Allergen | Non-Toxin | POSITIVE |
| DFGSIGGVFNSIGKA | HLA-DQA10301-DQB10301, HLA-DQA10501-DQB10301, HLA-DQA10501-DQB10302 | 0.6480 (ANTIGEN) | Non-Allergen | Non-Toxin | POSITIVE |
| GASGATWIDLVLEGG | HLA-DPA10103-DPB10402, HLA-DQA10101-DQB10501, HLA-DQA10301-DQB10302, HLA-DQA10401-DQB10402, HLA-DQA10501-DQB10201 | 0.6781 (ANTIGEN) | Non-Allergen | Non-Toxin | POSITIVE |
| KMEATELATVREYCY | HLA-DQA10102-DQB10602, HLA-DQA10201-DQB10202, HLA-DQA10301-DQB10302, HLA-DQA10401-DQB10402 | 0.9318 (ANTIGEN) | Non-Allergen | Non-Toxin | POSITIVE |
| LFGGMSWITQGLLGA | DRB1_0402, HLA-DQA10103-DQB10603, HLA-DQA10201-DQB10402, HLA-DQA10501-DQB10301, HLA-DQA10501-DQB10302 | 0.7270 (ANTIGEN) | Non-Allergen | Non-Toxin | POSITIVE |
| LGALLLWMGLQARDR | DRB1_0402, DRB1_1301, DRB1_1501, DRB1_1602, DRB4_0101, DRB4_0103, DRB5_0101, HLA-DQA10102-DQB10502, HLA-DQA10303-DQB10402, HLA-DQA10501-DQB10402, HLA-DQA10601-DQB10402 | 1.4396 (ANTIGEN) | Non-Allergen | Non-Toxin | POSITIVE |
| LVTVNPFISTGGANN | DRB1_1001, HLA-DQA10102-DQB10501, HLA-DQA10103-DQB10603, HLA-DQA10201-DQB10301, HLA-DQA10201-DQB10303, HLA-DQA10201-DQB10402, HLA-DQA10501-DQB10303 | 0.5279 (ANTIGEN) | Non-Allergen | Non-Toxin | POSITIVE |
| MKMEATELATVREYC | HLA-DQA10102-DQB10602, HLA-DQA10201-DQB10202, HLA-DQA10301-DQB10302, HLA-DQA10401-DQB10402 | 0.8154 (ANTIGEN) | Non-Allergen | Non-Toxin | POSITIVE |
| MSWITQGLLGALLLW | DRB1_0402, DRB1_1201, HLA-DPA10103-DPB10201, HLA-DQA10201-DQB10301, HLA-DQA10201-DQB10303 | 0.5673 (ANTIGEN) | Non-Allergen | Non-Toxin | POSITIVE |
| NLPWTSPATTDWRNR | HLA-DQA10201-DQB10402, HLA-DQA10303-DQB10402, HLA-DQA10501-DQB10302, HLA-DQA10601-DQB10402 | 1.4470 (ANTIGEN) | Non-Allergen | Non-Toxin | POSITIVE |
| PQAPSFTANMGEYGT | DRB3_0202, HLA-DQA10103-DQB10603, HLA-DQA10501-DQB10302 | 0.6128 (ANTIGEN) | Non-Allergen | Non-Toxin | POSITIVE |
| PTLDFKVMKMEATEL | DRB1_0101, DRB1_0103, DRB1_0401, DRB1_0405, DRB1_0701, DRB1_0801, DRB1_0901, DRB1_1001, DRB1_1101, DRB1_1201, DRB1_1602, DRB4_0101, HLA-DPA10103-DPB10402, HLA-DPA10103-DPB10601, HLA-DQA10201-DQB10402, HLA-DQA10501-DQB10201 | 0.9687 (ANTIGEN) | Non-Allergen | Non-Toxin | POSITIVE |
| REYCYEATLDTLSTV | DRB1_0401, DRB1_0405, HLA-DPA10103-DPB10601, HLA-DPA10103-DPB10201, HLA-DQA10201-DQB10202 | 0.6329 (ANTIGEN) | Non-Allergen | Non-Toxin | POSITIVE |
| SGINTEDYYVFTVKE | HLA-DPA10103-DPB10201, HLA-DQA10104-DQB10503, HLA-DQA10301-DQB10302, HLA-DQA10401-DQB10402 | 0.8979 (ANTIGEN) | Non-Allergen | Non-Toxin | POSITIVE |
| TKQTVVALGSQEGAL | DRB1_0101, HLA-DQA10103-DQB10603, HLA-DQA10201-DQB10301, HLA-DQA10201-DQB10303, HLA-DQA10501-DQB10303 | 0.8495 (ANTIGEN) | Non-Allergen | Non-Toxin | POSITIVE |
| TTQINYHWHKEGSSI | HLA-DQA10104-DQB10503, HLA-DQA10601-DQB10402 | 0.7808 (ANTIGEN) | Non-Allergen | Non-Toxin | POSITIVE |
| TIDCEARSGINTEDY | HLA-DQA10301-DQB10302, HLA-DQA10401-DQB10402 | 0.9033 (ANTIGEN) | Non-Allergen | Non-Toxin | POSITIVE |

**Supplementary Table 9: Final CTL and HTL epitopes selected for vaccine construct, and similarity percentage by sequence conservation analysis**

| **Epitope symbol** | **Epitope** | **DENV** | **ZIKV** | **YFV** | **WNV** | **JEV** |
| --- | --- | --- | --- | --- | --- | --- |
| **CTL epitopes** | | | | | | |
| CTL1 | NIKYEVAIF | NS | NS | NS | 100% | NS |
| CTL2 | TISPQAPSF | 87.50% | NS | NS | NS | NS |
| CTL3 | RDRSISLTL | NS | NS | NS | 88.89% | NS |
| CTL4 | QRVVFVIML | NS | NS | NS | 88.89% | 77.78% |
| CTL5 | RVVFVIMLM | NS | NS | NS | 77.78% | 66.67% |
| **HTL epitopes** | | | | | | |
| HTL1 | ALAIGWMLGSNNTQR | NS | NS | NS | 91.67% | 84.62% |
| HTL2 | DFGSIGGVFNSIGKA | NS | NS | NS | NS | 100% |
| HTL3 | GASGATWIDLVLEGG | NS | NS | NS | 92.86% | 93.33% |
| HTL4 | KMEATELATVREYCY | NS | NS | NS | 78.57% | 84.62% |
| HTL5 | LFGGMSWITQGLLGA | NS | NS | NS | 100% | NS |
| HTL6 | LGALLLWMGLQARDR | NS | NS | NS | 86.67% | NS |
| HTL7 | LVTVNPFISTGGANN | NS | NS | NS | 78.57% | 71.43% |
| HTL8 | MKMEATELATVREYC | NS | NS | NS | 73.33% | NS |
| HTL9 | MSWITQGLLGALLLW | NS | NS | NS | 100% | NS |
| HTL10 | NLPWTSPATTDWRNR | NS | NS | NS | 80% | 85.71% |
| HTL11 | PQAPSFTANMGEYGT | NS | NS | NS | 71.43% | NS |
| HTL12 | PTLDFKVMKMEATEL | NS | NS | NS | 73.33% | NS |
| HTL13 | REYCYEATLDTLSTV | NS | 87.50% | NS | 64.29% | 60% |
| HTL14 | SGINTEDYYVFTVKE | NS | NS | NS | 69.23% | 69.23% |
| HTL15 | TKQTVVALGSQEGAL | NS | NS | NS | 93.33% | 93.33% |
| HTL16 | TTQINYHWHKEGSSI | NS | NS | NS | 92.31% | NS |
| HTL17 | TIDCEARSGINTEDY | NS | NS | NS | 66.67% | 84.62% |

**Note:** DENV represents Dengue virus 1 (taxid:11053), ZIKV represents Zika virus (taxid:64320), YFV represents Yellow fever virus (taxid:11089), WNV represents West Nile virus (taxid:11082), JEV represents Japanese encephalitis virus (taxid:11072). NS represents No significant similarity found.

**
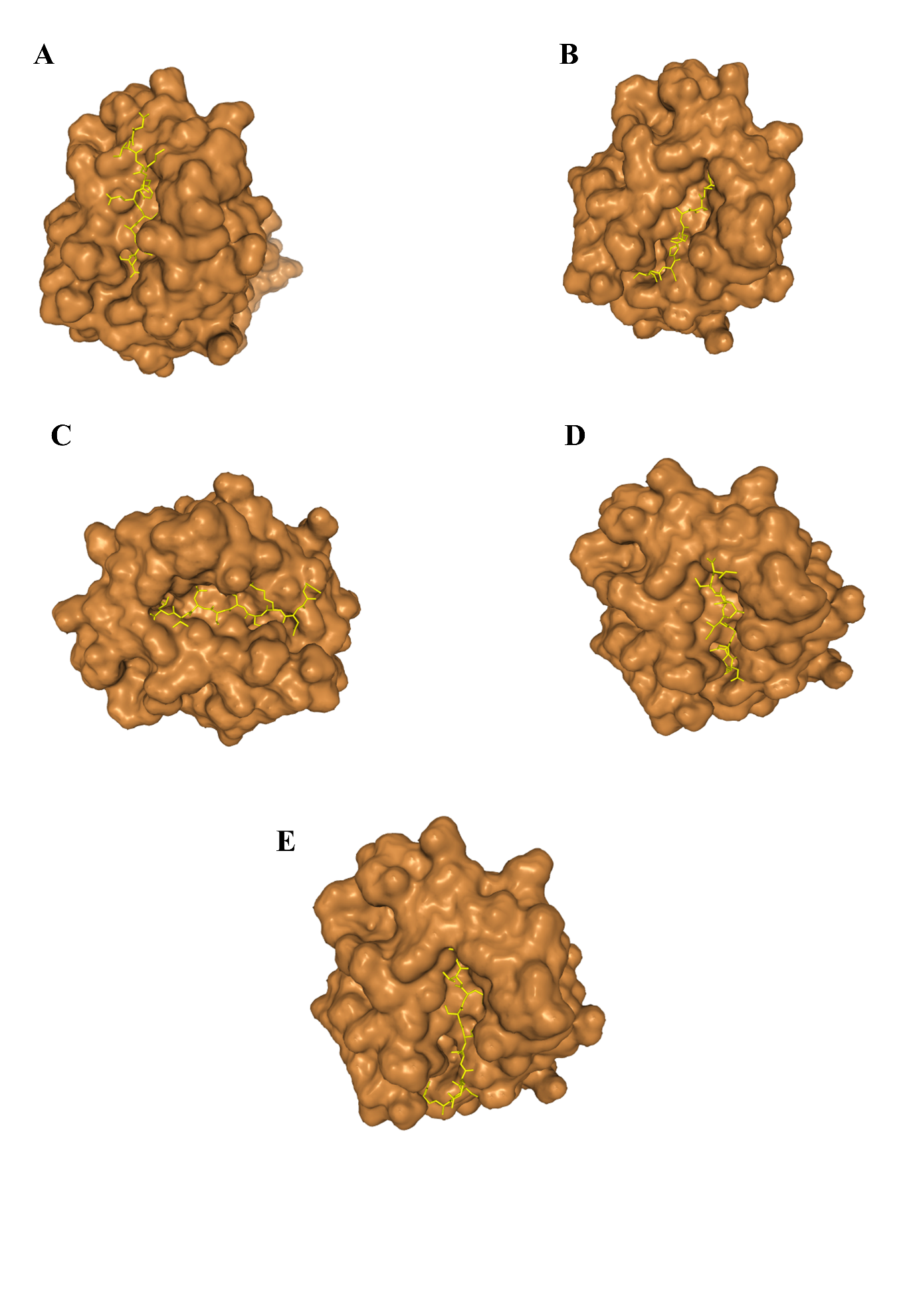
**

**Supplementary Figure S1:** Docked complexes of the CTL epitopes with HLA-A*02:01. The docked complexes of CTL1 (A), CTL2 (B), CTL3 (C), CTLA(D) and CTL5 (DE) against HLA-A*02:01 were shown.

**
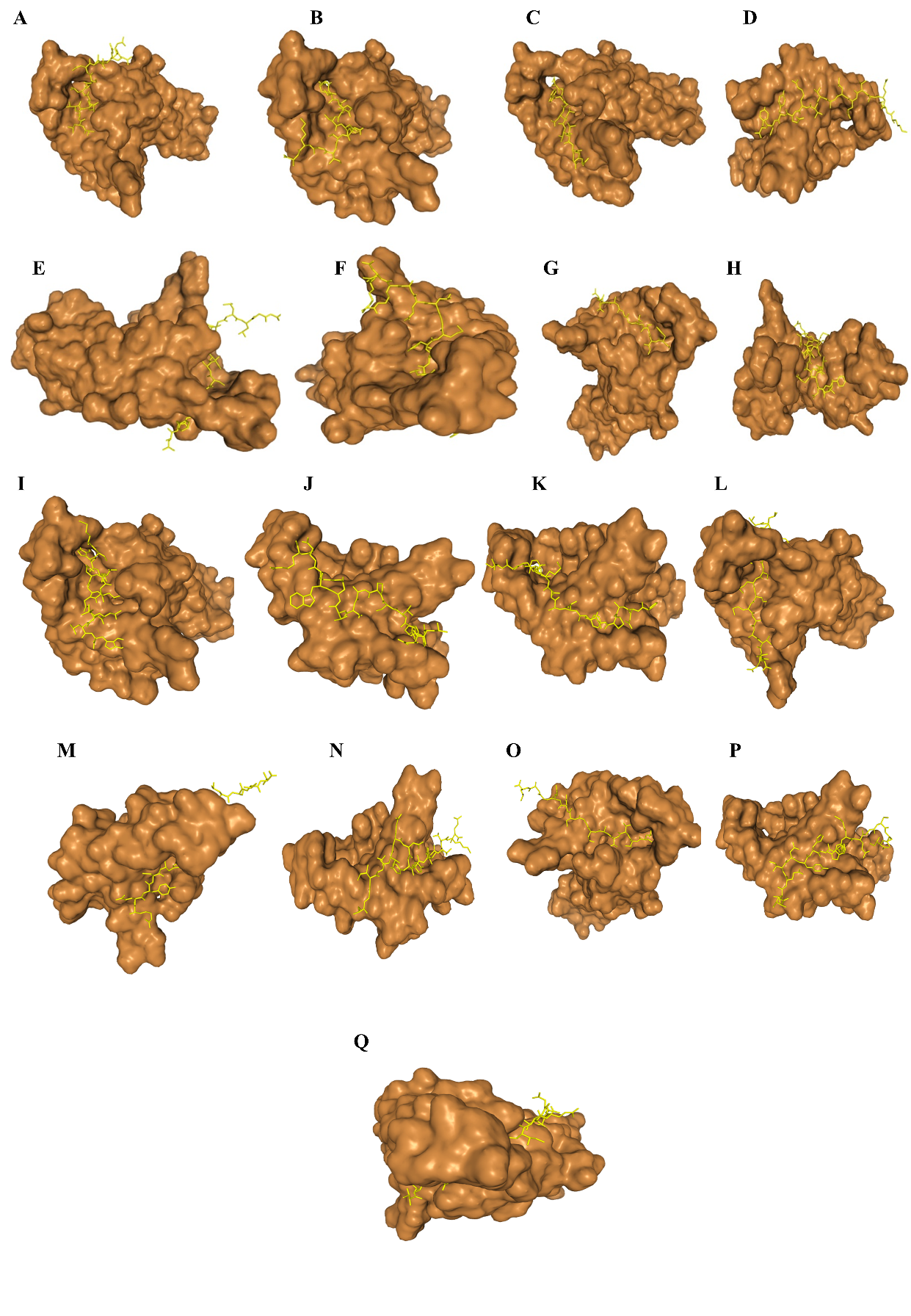
**

**Supplementary Figure S2:** Docked complexes of the HTL epitopes with HLA- DRB1*01:01. The docked complexes of HTL1 (A), HTL2 (B), HTL3 (C), HTL4 (D), HTL5 (E), HTL6 (F), HTL7 (G), HTL8 (H), HTL9 (I), HTL10 (J), HTL11 (K), HTL12 (L), HTL13 (M), HTL14 (N), HTL15 (O), HTL16 (P) and HTL17 (Q) against HLA- DRB1*01:01 were shown.
